# Two Genomic Loci Control Three Eye Colors in the Domestic Pigeon (*Columba livia*)

**DOI:** 10.1093/molbev/msab260

**Published:** 2021-08-30

**Authors:** Emily T Maclary, Bridget Phillips, Ryan Wauer, Elena F Boer, Rebecca Bruders, Tyler Gilvarry, Carson Holt, Mark Yandell, Michael D Shapiro

**Affiliations:** 1 School of Biological Sciences, University of Utah, Salt Lake City, UT, USA; 2 Department of Human Genetics and Utah Center for Genetic Discovery, University of Utah, Salt Lake City, UT, USA

**Keywords:** comparative genomics, QTL mapping, pigeon, iris color, pigment

## Abstract

The iris of the eye shows striking color variation across vertebrate species, and may play important roles in crypsis and communication. The domestic pigeon (*Columba livia*) has three common iris colors, orange, pearl (white), and bull (dark brown), segregating in a single species, thereby providing a unique opportunity to identify the genetic basis of iris coloration. We used comparative genomics and genetic mapping in laboratory crosses to identify two candidate genes that control variation in iris color in domestic pigeons. We identified a nonsense mutation in the solute carrier *SLC2A11B* that is shared among all pigeons with pearl eye color, and a locus associated with bull eye color that includes *EDNRB2*, a gene involved in neural crest migration and pigment development. However, bull eye is likely controlled by a heterogeneous collection of alleles across pigeon breeds. We also found that the *EDNRB2* region is associated with regionalized plumage depigmentation (piebalding). Our study identifies two candidate genes for eye colors variation, and establishes a genetic link between iris and plumage color, two traits that vary widely in the evolution of birds and other vertebrates.

## Introduction

A wide variety of genetic and developmental mechanisms influence evolutionary diversity in pigment type and patterning in the vertebrate epidermis, including epidermal appendages such as hair and feathers (Hoekstra 2006; Kelsh et al. 2009; Hubbard et al. 2010; Kaelin and Barsh 2013; Domyan et al. 2014; Parichy and Spiewak 2015; Bruders et al. 2020; Inaba and Chuong 2020). Pigments are also deposited in nonepidermal tissues in vertebrates, including the iris of the eye. Among vertebrate species, iris color varies widely. Some species have conspicuously colored bright yellow, red, or white irises, whereas others have dark irises. Iris coloration may be an evolutionarily adaptive trait that, like epidermal coloration, plays roles in crypsis and communication. For example, iris color is correlated with habitat in mantellid frogs, with arboreal species more likely to have bright eyes (Amat et al. 2013). Bright irises probably evolved multiple times in arboreal mantellid species, indicating that this trait might be adaptive. Iris color may be adaptive in birds as well. In owls, dark iris color likely coevolved with nocturnal behavior (Passarotto et al. 2018), whereas the bright white irises of jackdaws communicate that nesting sites are occupied (Davidson et al. 2014, 2017).

The genetic and developmental origins of variation in iris pigmentation are poorly understood. Although iris color varies widely among species, variability in iris color is limited within most species (Negro et al. 2017). However, intraspecific variation in iris color has evolved in humans and certain domestic species (Negro et al. 2017). In mammals, iris colors typically include shades of brown, green, and blue. These colors all arise from varying concentrations and deposition patterns of melanin pigments in the iris (Edwards et al. 2016). In contrast, the diversity of eye colors in amphibians and birds also depends on the presence of nonmelanin pigments. In birds, brilliant reds, oranges, and yellows arise from multiple nonmelanin pigment types, including pteridines, purines, and carotenoids (Oliphant 1987a).

**Fig. 1.**
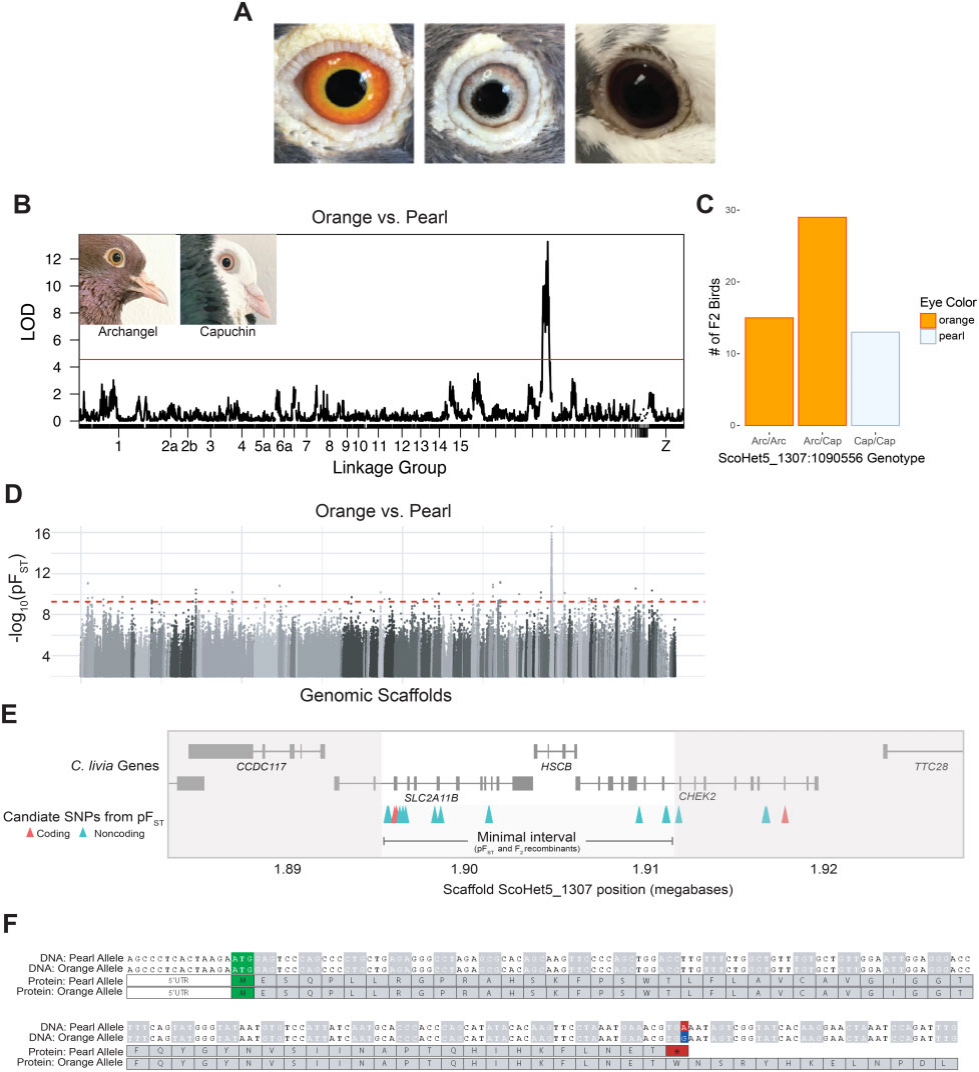
A single genomic locus is associated with pearl iris color in domestic pigeons. (*A*) Domestic pigeons typically have one of three major iris colors: the wild-type orange, pearl, or bull. (*B*) Genome-wide QTL scan for pearl eye in the Archangel x Old Dutch Capuchin cross. Red line indicates 5% genome-wide significance threshold. Insets: Archangel (left) and Capuchin (right) founders. (*C*) Eye color phenotypes of F_2_ progeny with different genotypes at the QTL peak marker. Arc, allele from the Archangel founder. Cap, allele from the Capuchin founder. (*D*) Whole-genome pF_ST_ comparisons of orange-eyed and pearl-eyed pigeons. Gray dots represent SNPs, with different shades indicating different genomic scaffolds. Dashed red line indicates genome-wide significance threshold. (*E*) Genomic context of the pearl eye candidate region. Gene models for the region are shown in gray. SNPs in coding regions are shown in red, SNPs in noncoding regions are shown in blue. The minimal candidate interval, bounded on the left by the end of the haplotype identified by pF_ST_ and on the right by F_2_ recombinants (see supplementary fig. S1, Supplementary Material online) is defined by gray shading. (*F*) Alignment of DNA (top) and predicted protein (bottom) sequences of SLC2A11B for pearl-eyed and orange-eyed pigeons. The start codon is highlighted in green. The DNA polymorphism at position ScoHet5_1307:1895934 is marked in red (pearl allele) or blue (orange allele); the resulting stop codon in the pearl allele is highlighted in red.

The domestic pigeon, *Columba livia*, shows intraspecific iris color variation among its 300+ different breeds. This variation, coupled with extensive genetic resources, makes the pigeon an ideal model to understand the genetics of iris pigmentation. Pigeons have three main iris colors: orange, pearl (white), and bull (dark brown; fig. 1*A*). Orange iris color is the ancestral state (Bond 1919), and “orange” eyes in actuality range in shades from yellow to red, depending on the density of blood vessels in the eye (Hollander and Owen 1939; Sell 2012). The pearl iris color is white, with tinges of pink and red from blood vessels. Lastly, the bull iris color is named based on its similarity in color to dark bovine eyes, and ranges from dark brown to almost black (Hollander and Owen 1939; Levi 1986). Breeding experiments show that the switch between the ancestral orange and derived pearl eye color is controlled by a single autosomal locus, and that orange is dominant to pearl (Bond 1919). Less is known about the inheritance of bull eye color. Although orange and pearl irises can be found in a variety of pigeon breeds, the bull iris color is primarily found in birds with white plumage (Hollander, 1939). Breeders have also reported birds with a phenotype known as “odd eyes” (Levi 1986; Sell 2012), in which one iris is a dark bull color and the other is either orange or pearl, suggesting that bull eye color may have a stochastic component.

Pigeons have two types of nonmelanin pigments in the iris: guanidines and pteridines (Oliphant 1987a). Guanidines are whitish opaque pigments, and pteridines are yellow-orange pigments (Oliphant 1987b). In orange-eyed pigeons, both guanidine and pteridine pigments are present in the iris stroma, whereas in white-eyed pigeons, only guanidine pigment is present (Oliphant 1987a, 1987b). In bull-eyed pigeons, both white and orange pigments are absent from the iris stroma, so the underlying dark melanin pigment of the iris pigment epithelium is not obscured (Bond 1919; Oliphant 1987b). The genetic and developmental mechanisms underlying loss of pteridine iris pigment in pearl-eyed birds or both pteridine and guanidine pigment in bull-eyed birds are currently unknown. Loss could arise from defects in pigment production or failure to transport the pigment into the iris stroma, for example.

To better understand the genetic mechanisms that control iris color in domestic pigeons, we used a combination of genomic mapping and laboratory crosses to identify two loci that are associated with eye color. We identified a nonsense mutation that segregates with pearl eye color and a second locus associated with bull eye color. We also found a genetic link between iris and feather color in birds with an array of plumage depigmentation phenotypes collectively known as piebalding, thereby establishing a genetic link that explains the anecdotal co-occurrence of iris and plumage color traits.

## Results

### A Single Genomic Locus Is Associated with Pearl Eye Color in the Domestic Pigeon

We used quantitative trait locus (QTL) mapping in an F_2_ intercross between an orange-eyed Archangel domestic pigeon and a pearl-eyed Old Dutch Capuchin domestic pigeon to identify loci associated with pearl eye color. In this cross, F_2_ progeny had either two pearl eyes (*n* = 12), one pearl eye and one bull eye (*n* = 1), two orange eyes (*n* = 40), or one orange eye and one bull eye (*n* = 5). We used a binary QTL model to compare birds with at least one pearl eye to birds with at least one orange eye, and identified a significant association between eye color and genotypes on linkage group 20 (fig. 1*B* and supplementary fig. S1, Supplementary Material online; peak marker ScoHet5_1307:1090556, peak LOD score = 13.28, genome-wide significance threshold 4.74, candidate region—defined as a 2-LOD interval from the peak marker—spans ScoHet5_149:3706619–ScoHet5_1307:1911647). The genotype at marker ScoHet5_1307:1090556 is perfectly associated with eye color in the cross, and all pearl-eyed F_2_ birds are homozygous for the pearl-eyed Capuchin allele (fig. 1*C*). This lone genome-wide peak is consistent with breeder reports that pearl eye is a monogenic trait with a simple recessive inheritance pattern.

In conjunction with QTL mapping, we compared whole-genome sequences from a diverse panel of orange-eyed (*n* = 28 from 17 domestic breeds and feral pigeons) and pearl-eyed pigeons (*n* = 33 from 25 breeds and ferals) using a probabilistic measure of allele frequency differentiation (pF_ST_; Kronenberg 2015; Domyan et al. 2016; Garrison et al. 2021; fig. 1*D*). We identified a single, 1.5-Mb genomic region on scaffold ScoHet5_1307 that was significantly differentiated between orange-eyed and pearl-eyed birds (ScoHet5_1307:1490703-3019601, genome-wide significance threshold pF_ST_ = 5.4 × 10^−^^10^, peak SNP pF_ST_ = 1 × 10^−16^, the minimum possible value with this data set). Because pearl eye color is recessive to orange, we searched for SNPs within the pF_ST_ and QTL peak regions to identify polymorphisms where pearl-eyed birds were always homozygous for the reference allele (the Danish Tumbler pigeon sequenced for the Cliv_2.1 reference assembly had pearl eyes; Shapiro et al. 2013; Holt et al. 2018; supplementary fig. S1*A*, Supplementary Material online). We identified 20 SNPs spanning a 22-kb region (ScoHet5_1307:1895516-1917937) that showed the expected segregation pattern between orange-eyed and pearl-eyed birds. This haplotype defined by sequencing overlaps partially with the 2-LOD interval defined by QTL mapping; thus, two independent approaches identified the same genomic region as a candidate for pearl eye.

To define the minimal region associated with pearl eye color, we examined the overlap between the QTL 2-LOD interval, individual informative F_2_ recombinants from the cross used for QTL mapping, and pF_ST_ results (supplementary fig. S1*B*, Supplementary Material online). By overlaying QTL and pF_ST_ results, we identified a 16-kb minimal region spanning ScoHet5_1307: 1895516-1911647 with 16 SNPs that were perfectly associated with pearl eye color (fig. 1*E*). Fourteen of these SNPs are intronic or intergenic, and two are coding mutations in the gene *SLC2A11B*.

### Pearl-Eyed Birds Harbor a Premature Stop Codon in Solute Carrier *SLC2A11B*

*SLC2A11B* is a strong candidate gene for pearl eye color. This gene has orthologs in fish and sauropsids, but not in mammals. Data from fish orthologs suggest that *SLC2A11B* plays a role in pigmentation. In medaka, for example, *SLC2A11B* is involved in the differentiation of pteridine-containing leucophore and xanthophore cells in scales (Kimura et al. 2014). The most closely related mammalian gene appears to be *SLC2A11* (*GLUT11*), which encodes a glucose transporter (Doege et al. 2001; Kimura et al. 2014).

To evaluate variants in the pearl eye candidate region, we first assessed the predicted impact of the two coding mutations in *SLC2A11B.* The first coding mutation (position ScoHet5_1307: 1895934) changes a tryptophan (orange allele) to a premature stop codon (pearl allele; fig. 1*F*). The second coding mutation (position ScoHet5_1307:1896042) results in a synonymous substitution 36 amino acids downstream of the premature stop codon.

The premature stop codon in pearl-eyed pigeons falls in exon 3 of *SLC2A11B*, and is predicted to severely truncate the resulting protein from 504 to 57 amino acids. Translation initiation at the next in-frame methionine would produce a protein missing the first 95 amino acids, but with the remaining 81% (409 of 504 amino acids) of the protein intact. To predict if such a truncated protein would be functional, we used InterProScan (Zdobnov and Apweiler 2001) and Phobius (Käll et al. 2004) to identify transmembrane domains and conserved functional motifs within the SLC2A11B protein, and examined sequence similarity across species (supplementary fig. S2*A*–*C*, Supplementary Material online). The first 94 amino acids of SLC2A11B are predicted to code for two transmembrane domains that are highly conserved. We used PROVEAN to predict the functional consequences of removing these domains and found that this truncation is expected to be detrimental to protein function (PROVEAN score of −189.145; Choi et al. 2012; Choi and Chan 2015). Therefore, the pearl mutation, which results in a loss of pteridines in the iris, is predicted to truncate a highly conserved protein that is associated with the differentiation of pteridine-containing pigment cells. The first two transmembrane domains of the SLC2A11B protein are highly conserved across species, yet we identified orthologs in two bird species, hooded crow (NCBI accession XP_019140832.1) and wire-tailed manakin (NCBI accession XP_027569903.1), in which the annotated protein sequence is missing the first of these transmembrane domains. Although we cannot rule out a misannotation in these genomes, neither species appears to have yellow-orange iris pigments. Hooded crows have dark eyes, whereas wire-tailed manakins have white irises (Madge 2020; Snow 2020), raising the possibility that neither species is capable of producing pteridine iris pigments due to a hypomorphic or null version of SLC2A11B. Thus, a gene associated with eye color variation in pigeons may be relevant to the evolution of iris color in birds more generally.

We additionally used targeted genotyping in F_2_ individuals from a cross between Racing Homer and Parlor Roller pigeon breeds, both of which can have either orange or pearl irises, to confirm the segregation of this nonsense mutation. We again found a perfect association between the nonsense SNP and pearl eye color (*n* = 25 F_2_ birds; *P *=* *2.24 × 10^−7^, Fisher’s exact test). Although the nonsense mutation in *SLC2A11B* is predicted to be highly damaging, we cannot fully rule out the role of intronic or intergenic SNPs, which may regulate gene expression. To evaluate the evolutionary conservation of intronic and intergenic sequences in the region, we performed alignments of syntenic regions across four nonavian vertebrates (three-toed box turtle, saltwater crocodile, American alligator, and painted turtle). We found that none of the noncoding SNPs within the pearl eye haplotype fall within conserved noncoding elements. Of the remaining SNPs in the haplotype identified by pF_ST_, but excluded based on F_2_ recombinants (fig. 1*E* and supplementary fig. S1*B*, Supplementary Material online), one variant results in a nondamaging synonymous substitution in *CHEK2*, a gene not known to be associated with pigmentation. The remaining variants in the pearl-eye haplotype are intronic or intergenic and do not reside within evolutionarily conserved regions. As a result, we conclude that the nonsense mutation in *SLC2A11B* is the strongest candidate for causing pearl eye color.

### Expression of the *SLC2A11B* Pearl Allele Is Reduced

Using high-throughput RNA-sequencing (RNA-seq) data sets, we found that *SLC2A11B* shows very low levels of expression in most adult tissues, including the retina, but substantial expression in both Hamburger-Hamilton stage 25 (HH25; Hamburger and Hamilton 1951) whole embryos (*n* = 2) and embryo heads (*n* = 12) (supplementary fig. S3*A*–*D*, Supplementary Material online). Based on genotypes at the two coding SNPs in the pearl eye haplotype, we found that gene expression in embryo head samples only shows changes associated with the pearl eye haplotype in *SLC2A11B*, and not in any other genes within 15 kb of the pearl eye haplotype, further supporting *SLC2A11B* as the primary candidate for the pearl eye phenotype (supplementary fig. S3*A*, Supplementary Material online). Embryo heads homozygous for the pearl allele show a significant reduction in *SLC2A11B* expression (*P *=* *3.94 × 10^−6^, two-tailed *t*-test; supplementary fig. S3*B*, Supplementary Material online). Analysis of read distribution within the *SLC2A11B* gene shows a decrease in spliced reads specifically within the first three annotated exons, suggesting that alternative splicing or nonsense-mediated decay may be occurring (supplementary fig. S3*D*, Supplementary Material online). In contrast, we would anticipate that alterations in expression due to regulatory changes would result in a more uniform decrease of reads across the entire transcript. In summary, the pearl eye phenotype is associated with a nonsense mutation in a known mediator of yellow-orange pigments, which in turn is linked to a significant decrease in *SLC2A11B* expression, possibly due to nonsense-mediated decay of the mutant transcripts.

### QTL Mapping Identifies a Single Genomic Locus Associated with Bull Eye Color

Variation at *SLC2A11B* appears to act as a switch between two of the major pigeon iris colors, orange and pearl eye, but it does not explain the third major color, bull eye. Bull eyes are dark brown, lacking both orange and white pigment in the iris. However, pigeon breeders observe that bull eye color can occur on either an orange or pearl genetic background (Sell 2012), suggesting that the loss of orange pigment in bull eyes likely arises from a mechanism that does not involve *SLC2A11B*.

**Fig. 2.**
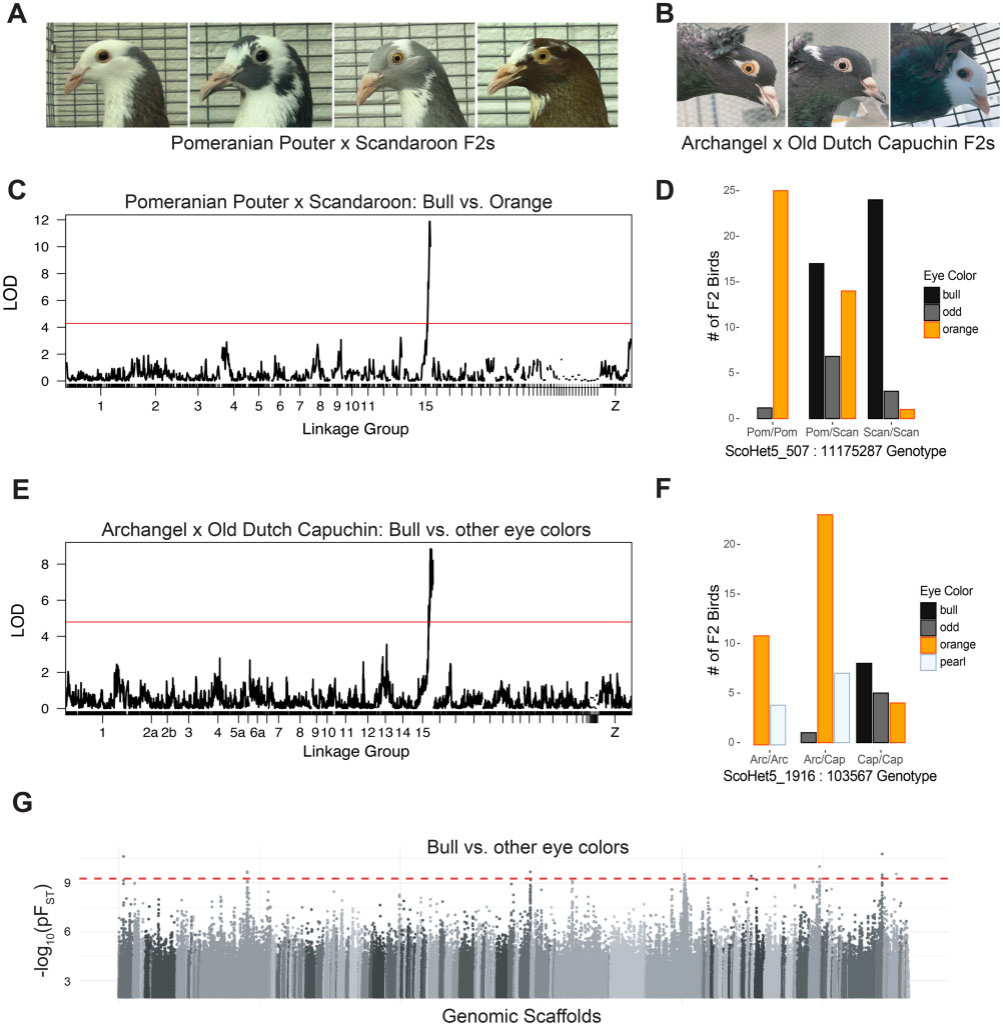
A single genomic locus is associated with bull eye color in two F_2_ intercrosses. (*A*) F_2_ offspring from an intercross between a Pomeranian Pouter and a Scandaroon have either bull (left two images) or orange (right two images) eyes. (*B*) F_2_ offspring from an intercross between an Archangel and an Old Dutch Capuchin have orange (left), pearl (center), or bull (right) eyes. (*C*) Genome-wide QTL scan of the Pomeranian Pouter x Scandaroon cross for bull eye. Red line indicates 5% genome-wide significance threshold. (*D*) Iris color phenotype counts for each genotype at the bull eye peak marker from the Pomeranian Pouter x Scandaroon cross. Pom, allele from Pomeranian Pouter founder. Scan, allele from Scandaroon founder. (*E*) Genome-wide QTL scan of the Archangel x Old Dutch Capuchin cross for bull eye. Red line indicates 5% genome-wide significance threshold. (*F*) Iris color phenotype counts for each genotype at the bull eye peak marker from the Archangel x Capuchin cross. Arc, allele from the Archangel founder. Cap, allele from the Capuchin founder. (*G*) Whole-genome pF_ST_ comparisons of bull-eyed birds to birds with nonbull (orange or pearl) eyes. Dashed red line indicates 5% threshold for genome-wide significance.

To identify the genetic basis of bull eye color, we used QTL mapping in two independent F_2_ intercrosses. In a cross between orange-eyed Pomeranian Pouter and bull-eyed Scandaroon pigeon breeds, F_2_ birds had either two bull eyes (*n* = 41), two orange eyes (*n* = 40), or “odd eyes,” where one eye has a pigmented iris stroma and the other eye is bull (*n* = 12) (fig. 2*A*). Using a binary model where odd-eyed birds were included in the “bull eye” group, we identified a QTL on linkage group 15 (fig. 2*C* and supplementary fig. S4, Supplementary Material online; peak marker ScoHet5_507:11175287, LOD score = 11.89, genome-wide significance threshold = 4.28). The peak region spans 2.0 Mb across two genomic scaffolds, from ScoHet5_507:9736663 to ScoHet5_683.1:279252, and includes 42 annotated genes. Nearly all birds with two copies of the Scandaroon allele have bull eyes (*n* = 24) or odd eyes (*n* = 3), whereas heterozygotes show a mix of phenotypes (fig. 2*D*), indicating this phenotype may have a stochastic component, incomplete penetrance, or modifiers that we were unable to map.

In the cross between the orange-eyed Archangel and the pearl-eyed Old Dutch Capuchin that we originally used to map pearl eyes, neither founder had the bull eye phenotype. However, some offspring had either two bull eyes (*n* = 8) or odd eyes (*n* = 6) (fig. 2B). We used a binary model to compare these 14 birds with at least one bull eye to 52 F_2_ birds without bull eyes (either two orange or two pearl eyes). Here, too, we identified a single locus associated with bull eye color on linkage group 15 (fig. 2*E* and supplementary fig. S4, Supplementary Material online; peak marker ScoHet5_1916:103567, peak LOD score = 8.85, genome-wide significance threshold = 4.61). The peak region spans 1.5 Mb across eight scaffolds, including the same two scaffolds identified in the Scandaroon x Pomeranian Pouter cross, and captures 44 annotated genes. The additional scaffolds within this QTL lack informative markers in the Pomeranian Pouter x Scandaroon cross. Although the Old Dutch Capuchin founder does not have bull eyes, nearly all bull-eyed and odd-eyed F_2_s carry two copies of the Capuchin allele at the peak marker (fig. 2*F*). This suggests that, unlike in the Pomeranian Pouter x Scandaroon cross, inheritance of bull eye color in the Archangel x Capuchin cross is recessive with low penetrance. The lone odd-eyed bird in the latter cross is heterozygous for the Capuchin allele at ScoHet5_1916:103567 and may have a recombination event between the peak QTL marker and the causative bull eye variant. In both crosses, excluding odd-eyed birds from the data set does not substantially alter the QTL regions, though the 2-LOD interval for the Pomeranian Pouter x Scandaroon cross is slightly smaller when these individuals are excluded (supplementary fig. S4*A* and *B*, Supplementary Material online).

### Bull Eye Color and Allelic Heterogeneity

QTL mapping identified a single locus associated with bull eye color in two different crosses. Although the interval boundaries vary between the two crosses, the intervals identified in both crosses are broadly overlapping (supplementary fig. S4*C*, Supplementary Material online). Despite this broad overlap, the inheritance pattern of bull eye appears to differ in each case (fig. 2*D* and *F*). In an attempt to account for these differences, we performed additional QTL scans conditioning on the genotype at the peak markers identified by our initial analysis to look for modifier loci that might be specific to each cross. However, we did not identify any additional loci that reached the threshold for statistical significance for additive or interactive models. Furthermore, genome-wide pF_ST_ analysis comparing bull-eyed birds (*n* = 18) to a background data set of both orange-eyed and pearl-eyed birds (*n* = 61) identified a small number of differentiated SNPs across multiple scaffolds, including ScoHet5_507, but did not pinpoint a single well-differentiated region shared all bull-eyed breeds (fig. 2*G*). Together, these results imply that, although our QTL mapping identified the same genomic region in two separate crosses, the variants that give rise to bull eye color are probably not the same across all pigeon breeds.

### Bull Eye Color Is Associated with Plumage Depigmentation

**Fig. 3.**
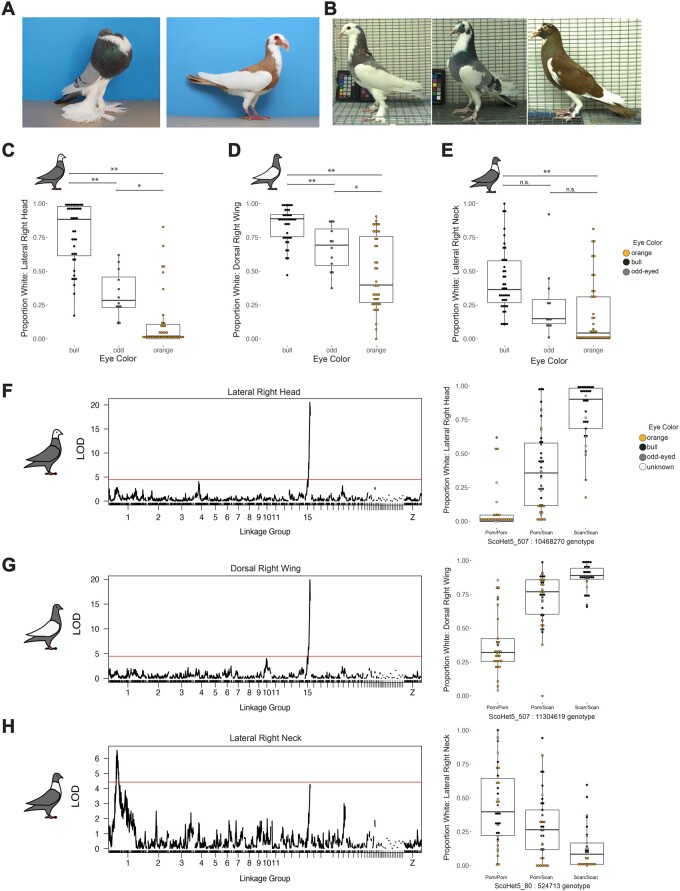
Bull eye color is associated with white plumage in an F_2_ intercross. (*A*) Examples of standard plumage patterning for the Pomeranian Pouter (left) and Scandaroon (right) breeds. Photos by Layne Gardner, used with permission. (*B*) Examples of variable piebald plumage patterning in Pomeranian Pouter x Scandaroon F_2_ offspring. (*C*–*E*) Boxplots of association between eye color and proportion of white plumage on the (*C*) lateral right head, (*D*) dorsal right wing, and (E) lateral right neck of F_2_ birds. **, *P *≤* *0.0001; *, 0.0001 < *P *≤* *0.01; n.s., *P *>* *0.01. Boxes span from the first to third quartile of each data set, with lines indicating the median. Whiskers span up to 1.5× the interquartile range. (*F*–*H*) QTL scans for proportion of white plumage (left side of the panel) and proportion of white plumage by genotype at the peak marker (right) for (*F*) lateral right head, (*G*) dorsal right wing, and (*H*) lateral right neck. Red line, 5% genome-wide significance threshold.

Pigeon hobbyists have long noted that bull eye color is most common in birds with white plumage (Sell 2012), including individuals with solid white plumage and those with a range of piebalding phenotypes. Piebalding is characterized by broad regions of white and pigmented feathers, and these regionalized depigmentation patterns are often breed specific. Both the Scandaroon and Pomeranian Pouter cross founders show breed-specific piebald patterning, and the F_2_ offspring of this cross show highly variable piebalding across multiple body regions (fig. 3*A* and *B*). We quantified the proportion of white plumage in 15 different body regions in the F_2_ progeny of the Pomeranian Pouter x Scandaroon cross and found that plumage color in many body regions is significantly associated with bull eye color in the Pomeranian Pouter x Scandaroon cross. The strength of this relationship varies by region, with areas like the lateral head and dorsal wing (fig. 3*C* and *D*) showing a stronger relationship with eye color than the lateral neck (fig. 3*E*; additional body regions are shown in supplementary fig. S5, Supplementary Material online).

To further evaluate the genetic relationship between piebalding and bull eye color, we used QTL mapping to identify two genomic regions associated with white plumage (fig. 3*F*–*H*, supplementary fig. S6, Supplementary Material online). Each locus is associated with white plumage in specific body regions and explains 15–58% of the variance in the cross (table 1). The QTL on linkage group 1 is associated with white plumage on the neck and dorsal body, and individuals with white plumage carry the Pomeranian Pouter allele at the linkage group 1 candidate locus. The QTL on linkage group 15 is associated with white plumage on the head, wings, and dorsal body; for this locus, the Scandaroon allele is associated with white plumage. The linkage group 15 piebalding QTL overlaps with the locus identified for bull eye (supplementary fig. S7*A*, Supplementary Material online), suggesting either closely linked variants in the same or different genes, or the same variant controlling both traits. These associations are consistent with breed-specific plumage patterns, as Scandaroon pigeons typically have white plumage on the head, wings, and ventral body, whereas the Pomeranian Pouter breed is characterized by a white “bib” on the neck (see examples in fig. 3*A*). In summary, at least two genetic loci control piebalding in pigeons, one of which overlaps with the bull eye locus, and these loci act in a regionally and breed-specific manner.

**Table 1.** Summary of QTL for Regional White Plumage.

Body Region	Linkage Group	Peak Marker	Peak LOD Score	PVE	Associated Allele
Dorsal right wing	15	ScoHet5_507_11304619	19.9	57	Scan
Dorsal left wing	15	ScoHet5_507_11304619	15.5	49	Scan
Dorsal body	1	ScoHet5_80_11511249	9.11	30	Pom
Dorsal body	15	ScoHet5_683.1_42424	4.45	15	Scan
Dorsal neck	1	ScoHet5_80_3402497	5.72	22	Pom
Dorsal head	15	ScoHet5_507_11175287	22.4	63	Scan
Ventral right wing	15	ScoHet5_507_11175287	12.2	40	Scan
Ventral left wing	15	ScoHet5_507_11227444	9.34	33	Scan
Ventral body	15	ScoHet5_507_11058018	11.4	38	Scan
Ventral tail	15	ScoHet5_683.1_42424	17.4	52	Scan
Ventral neck	1	ScoHet5_80_524713	4.81	21	Pom
Ventral neck	15	ScoHet5_507_11175287	7.39	30	Scan
Lateral right head	15	ScoHet5_507_10468270	20.5	58	Scan
Lateral right neck	1	ScoHet5_80_524713	6.56	15	Pom
Lateral left head	15	ScoHet5_507_11058018	20.7	58	Scan
Lateral left neck	1	ScoHet5_2444_504541	4.37	17	Pom

Note.—PVE, percent variance explained; Pom, Pomeranian Pouter; Scan, Scandaroon.

### *EDNRB2* Is a Candidate Gene for Bull Eye Color and White Plumage

We next wanted to identify candidate genes for bull eye color and white plumage within the linkage group 15 QTL region. Of the 60 genes included in at least one of the Pomeranian Pouter x Scandaroon (2.0 Mb, 42 genes) or Archangel x Old Dutch Capuchin (1.5 Mb, 44 genes) bull eye QTL peaks, comparison to gene ontology databases did not identify any genes with GO annotations related to pigmentation. However, we were able to find potential links to pigment patterning for five genes (table 2).

**Table 2.** Summary of Pigment-Associated Genes within the LG15 QTL.

Gene Name	Scaffold	Position	Role in Pigmentation
*CDX1* ^a^	ScoHet5_507	10023919–10038223	Involved in neural crest development, reduction in *CDX1* is associated with white spotting in mice (Sanchez-Ferras et al. 2016)
*NSDHL*	ScoHet5_507	11023840–11035275	Mice with heterozygous mutations can have striped coats (Liu et al. 1999)
*VAMP7*	ScoHet5_507	11126494–11144082	SNARE protein involved in TYRP1 trafficking to the melanosome (Tamura et al. 2011)
*EDNRB2*	ScoHet5_507	11162676–11176857	Controls migration of neural crest-derived pigment cells (Pla et al. 2005; Harris et al. 2008); linked to plumage pigmentation phenotypes in multiple avian species (Miwa et al. 2007; Kinoshita et al. 2014; Li et al. 2015; Wu et al. 2017; Xi et al. 2020)
*GPR119* ^b^	ScoHet5_1916	115025–116006	G-protein-coupled receptor expressed in human melanocytes (Scott et al. 2006)

a Gene outside of Archangel x Capuchin 2-LOD interval.

b Gene on scaffold not present in Pomeranian Pouter x Scandaroon linkage map.

We assessed expression of all genes in the Pomeranian Pouter x Scandaroon and Archangel x Old Dutch Capuchin QTL intervals using RNA-seq data from whole HH25 embryo heads. Embryos were collected from pairs of Racing Homers (nonpiebald, orange or pearl irises) or Oriental Frills (piebald, bull irises). Although the adult iris color is not evident at this early stage, the offspring of two bull-eyed birds are expected to be bull eyed, and the Racing Homer pairs are not piebald or bull eyed. A small number of genes showed small (Log2-fold change ≤ 0.71) but significant differences in expression (supplementary table S1, Supplementary Material online). However, one gene, *EDNRB2*, showed a stark increase in expression in Racing Homers compared to Oriental Frills (Log2-fold change = 6.42; supplementary fig. S7*B* and table S1, Supplementary Material online).

*EDNRB2* plays a critical role in the migration of neural crest derived pigment cells (Harris et al. 2008). Migrating neural crest cells contribute to both the epidermal pigment and the iris stroma, providing a developmental link between plumage and iris color (Gage et al. 2005; Davis-Silberman and Ashery-Padan 2008). Although *EDNRB2* shows a drastic reduction in expression in piebald and bull-eyed Oriental Frill embryos, expression in Racing Homers is highly variable (supplementary fig. S7*C*, Supplementary Material online). This may be due to minor differences in staging. Mutations in *EDNRB2* are associated with depigmentation phenotypes in several domestic bird species, including “Panda” plumage in Japanese Quail, spot patterning in ducks, tyrosinase independent mottling in chickens, and white plumage with dark eye color in Minohiki chickens (Miwa et al. 2007; Kinoshita et al. 2014; Li et al. 2015; Xi et al. 2020). Additionally, changes in the mammalian orthologue *ENDRB* are responsible for piebalding phenotypes in mice, the piebald-like frame overo pattern in horses, and epidermal pigmentation changes in Waardenburg-Shah syndrome type IV in humans (Koide et al. 1998; Metallinos et al. 1998).

Both frame overo patterning and Waardenburg-Shah syndrome are also associated with changes in iris color: humans and horses with *EDNRB* mutations often have blue eyes, and heterochromia is commonly observed (Metallinos et al. 1998; Pingault et al. 2010; Issa et al. 2017). The *EDNRB* genomic locus is also associated with light eyes and heterochromia in pigs (Moscatelli et al. 2020). In mammals, the blue iris phenotype is caused by reduction of melanin pigment in the iris stroma (Metallinos et al. 1998; Sturm and Larsson 2009). Similarly, bull eye color in pigeons results from the loss of neural crest-derived iris stromal pigment; the eye appears dark because melanin pigments are no longer obscured by overlying guanidines and pteridines (Oliphant 1987b).

Given the known role of endothelin receptors in epidermal and iris coloration in other vertebrates, *EDNRB2* is a compelling candidate for the linked piebalding and bull eye phenotypes in domestic pigeons. It remains possible that plumage color and bull iris are influenced by two different closely-linked genes within the linkage group 15 QTL. Nevertheless, the links between iris and epidermal pigment in vertebrates with *EDNRB* mutations make a compelling case for *EDNRB2* as a candidate gene for both phenotypes in pigeons. We examined the allele frequencies and genotypes of SNPs within *EDNRB2* coding regions in both the bull-eyed and nonbull-eyed populations used for pF_ST_ analysis and did not identify any coding polymorphisms that were unique to bull-eyed birds, suggesting that noncoding regulatory changes may mediate bull eye color and piebalding in domestic pigeons. Due to the allelic heterogeneity and incomplete penetrance of the bull eye phenotype, however, we cannot rule out coding changes in *EDNRB2*, or other candidate genes within the region, as mediators of the bull eye phenotype.

## Discussion

### *SLC2A11B* and Pearl Eyes

Using comparative genomic and classical genetic approaches, we identified two candidate loci that control the three major iris colors of domestic pigeons. A locus on scaffold ScoHet5_1307 is associated with pearl eye color. This region contains a SNP fixed in pearl-eyed birds that changes a tryptophan to a premature stop codon in exon 3 of the solute carrier *SLC2A11B*, and was also recently identified by Andrade et al. and Si et al. as a candidate mutation for pearl eye color in pigeons (Si et al. 2021; Andrade et al. 2021). We found that the nonsense mutation is associated with pearl iris color in individually phenotyped pigeons from a wide array of domestic breeds, consistent with a single mutation arising early in domestication (Si et al. 2021). We also showed that the *SLC2A11B* locus is the one and only genomic region that segregates with pearl eye color in two F_2_ crosses. Our results support the trio genotyping of the *SLC2A11B* mutation performed by Andrade et al. (2021), and our linkage mapping excludes a role for the remainder of the genome in the switch between orange and pearl eyes. Intriguingly, although all pearl-eyed birds in our sample share a common *SLC2A11B* allele, pigeon breeders have also identified a second locus associated with white iris color that appears to be genetically distinct and is linked to brown plumage color (Levi 1986; Sell 2012). Future analysis of individual birds with this “false pearl” eye color could expand our understanding of the genes affecting pteridine synthesis and localization in the eyes of birds.

The *SLC2A11B* gene is not well characterized, but likely plays an evolutionarily conserved role in the development of pteridine-containing pigment cells. A nonsense mutation in *SLC2A11B* in medaka is associated with loss of mature pteridine-containing leucophores and xanthophores, and the Zebrafish Mutation Project identified differentiation defects in *SLC2A11B*-mutant xanthophores (Kimura et al. 2014). Si *et al.* (2021) additionally identified a frameshift mutation in *SLC2A11B* in cormorants, which have unique blue eyes and appear to lack pteridine pigments in the iris. Similarly, the missing transmembrane domain in the manakin and crow described here might render SLC2A11B incapable of pteridine deposition.

*SLC2A11B* does not have a mammalian ortholog, and its presence is restricted to species that have xanthophores or xanthophore-like cells (Kimura et al. 2014). Comparative analysis of solute carriers across species shows that the *SLC2A11B* gene likely originated prior to the teleost fish-specific genome duplication, and was then lost in mammals (Kimura et al. 2014). Loss of *SLC2A11B* may have restricted the repertoire of pigments that mammals can synthesize.

### Allelic Heterogeneity at the Bull Eye Locus

Observations by pigeon breeders previously indicated a simple recessive mode of inheritance for pearl eye color (Sell 2012), and this is confirmed by our analyses. The third major iris color in domestic pigeons, bull eye, appears to have a more complicated inheritance pattern. Through QTL mapping in two F_2_ crosses, we identified a single genomic locus on linkage group 15 that is associated with bull eye color. As previously noted by breeders, bull eye color is associated with white plumage (Sell 2012), and QTL mapping identified a strong association between the same linkage group 15 locus and piebald plumage patterning on the wing and head.

Despite the overlap in QTL for bull eye color in two F_2_ crosses and the QTL for white plumage, we were unable to pinpoint a single mutation within this locus associated with bull eye color through a comparative genomic approach. This suggests that bull eye may not be caused by a single genetic variant that is shared across breeds. Instead, the linkage group 15 QTL regions may harbor multiple breed-specific mutations. Complex and diverse regulatory changes at a single locus have previously been linked to pigment traits, for example, at the *Agouti* locus (Linnen et al. 2013). A similar situation in pigeons would provide a concise explanation for the association between bull eye color and multiple breed-specific piebald patterns; however, we cannot currently distinguish between changes in multiple closely linked genes and different changes in a single gene. Future work will examine the genetic underpinnings of regionalized plumage patterning in F_2_ crosses and work toward identification of specific genetic variants associated with bull eye color and the piebald plumage that typically accompanies it.

### *EDNRB2* and Constraints on Endothelin Receptor Evolution

Although the specific mutations that cause bull eye color and white plumage color remain unknown, the linkage group 15 QTL for bull eye color and piebalding contains a strong candidate gene, *EDNRB2.* The endothelin signaling pathway plays critical roles in the development and migration of multiple neural crest cell populations, including pigment cells. In mammals, mutations in the endothelin receptor *ENDRB* are linked to piebalding in mice; lethal white foal syndrome in horses; and Waardenburg Shah syndrome type 4A in humans, which is characterized by changes in hair, skin, and eye pigment, as well as congenital defects in enteric nervous system development (Read and Newton 1997; Koide et al. 1998; Metallinos et al. 1998; Jabeen et al. 2012). In several bird species, coding and regulatory variants of *EDNRB2* are associated with white plumage phenotypes and dark eye color (Miwa et al. 2007; Kinoshita et al. 2014; Li et al. 2015; Wu et al. 2017; Xi et al. 2020), but they are not typically linked to other major pathologies. Thus, although endothelin signaling is linked to pigmentation changes across vertebrates, *ENDRB* mutations in mammals are typically associated with deleterious pleiotropic effects, whereas *EDNRB2* mutations in birds are not.

The endothelin signaling pathway in vertebrates evolved through multiple rounds of gene duplication, and most bony vertebrates have three endothelin receptor genes: *EDNRA*, *EDNRB1*, and *EDNRB2* (Braasch, Volff, et al. 2009). Expression of different combinations of endothelin receptors and ligands characterize unique cell populations. In *Xenopus*, chicken, and quail, for example, *EDNRB2* is expressed specifically in migrating and postmigratory melanophores, whereas nonpigment neural crest populations, like skeletal and trunk neural crest cells, express *EDNRA* or *EDNRB1* (Square et al. 2016)*.* However, *EDNRB2* was lost in therian mammals, and the sole endothelin B receptor *ENDRB* is expressed in both trunk neural crest populations and melanophores (Braasch, Volff, et al. 2009; Square et al. 2016). As a result, in therian mammals, changes in endothelin signaling typically affect both pigmentation and neurogenesis. The retention of *EDNRB2* in nonmammalian vertebrates, on the other hand, may permit the evolution and development of novel pigment patterns because the genetic controls of pigment cell migration and neurogenesis are uncoupled.

### Gene Duplication and Retention May Mediate the Evolution of Pigment Diversity across Species

The retention of *EDNRB2* in nonmammalian vertebrates, and the diverse endothelin-mediated pigment patterns identified across bird species, point to a role for gene duplication in mediating or constraining diversity in both pigment type and patterning. In species that retained *EDNRB2*, subfunctionalization mediates the evolution of novel pigment patterns such as piebalding, whereas in species that lost *EDNRB2*, such changes are severely constrained by the requirement for a functional endothelin receptor B gene. This idea of gene loss restricting pigment phenotypes is also relevant to the retention of our pearl eye candidate gene *SLC2A11B*, which is only present in species with pteridine-containing xanthophore- or leucophore-like cells. Solute carriers in the *SLC2A* family also evolved through multiple rounds of gene duplication, though their evolutionary history is not as clear as that of endothelin ligands and receptors due to multiple segmental duplication events (Kimura et al. 2014; Lorin et al. 2018). Gene duplication and retention permitted the striking expansion and evolution of novel pigment types and patterns in teleost fish (Braasch, Brunet, et al. 2009; Lorin et al. 2018). The identification of *SLC2A11B* and *EDNRB2* as candidate genes for pigeon eye color suggests that similar patterns of retention of gene duplicates may mediate the evolution of pigment phenotypes in additional vertebrate species, including birds.

## Materials and Methods

### Animal Husbandry and Phenotyping of F_2_ Offspring

Pigeons were housed in accordance with protocols approved by the University of Utah Institutional Animal Care and Use Committee (protocols 10-05007, 13-04012, and 19-02011).

Two intercrosses were used in these studies. An intercross between a male Pomeranian Pouter and two female Scandaroons was performed to generate 131 F_2_ offspring (Domyan et al. 2016). An intercross between a male Archangel and a female Old Dutch Capuchin generated 98 F_2_ offspring.

### Whole-Genome Resequencing

DNA was extracted from blood samples collected with breeders’ written permission at the annual Utah Premier Pigeon Show or from our lab colony using the Qiagen DNEasy Blood and Tissue Kit (Qiagen, Valencia, California). Samples were treated with RNAse during extraction. Isolated DNA was submitted to the University of Utah High Throughput Genomics Shared Resource for library preparation using the Illumina Tru-Seq PCR-Free library kit. The resulting libraries were sequenced on either the Illumina HiSeq or Illumina NovaSeq platforms. Raw sequence data for 54 previously unpublished samples are available in the NCBI Sequence Read Archive under BioProject accession PRJNA680754. These data sets were combined with previously published data sets (BioProject accessions PRJNA513877, PRJNA428271, and PRJNA167554) for variant calling. Sequencing coverage varies by sample, sample IDs, and coverage are summarized in supplementary table S3, Supplementary Material online.

### Genomic Analyses

Variant calling was performed with FastQForward, which wraps the BWA short-read aligner and Sentieon (sentieon.com) variant calling tools to generate aligned BAM files (fastq2bam) and variant calls in VCF format (bam2gvcf). Sentieon is a commercialized variant calling pipeline that allows users to follow GATK best practices using the Sentieon version of each tool (Auwera et al. 2013). FastQForward manages distribution of the workload to these tools on a compute cluster to allow for faster data processing than when calling these tools directly, resulting in runtimes as low as a few minutes per sample.

A step-by-step summary of the workflow is available at support.sentieon.com/manual/DNAseq_usage/dnaseq/ (last accessed July 16, 2021). Briefly, raw sequencing reads from resequenced individuals were aligned to the Cliv_2.1 reference assembly (Holt et al. 2018) using fastq2bam, which utilizes the default settings of the BWA aligner. Reads are then deduplicated using samblaster. Variant calling was performed on 186 resequenced individuals, including 132 previously published samples (Shapiro et al. 2013; Domyan et al. 2016; Vickrey et al. 2018; Bruders et al. 2020), using bam2gvcf with the quality filter “–min_base_qual 20”, and individual genome variant call format (gVCF) files were created. Joint variant calling was performed on all 186 individuals using the Sentieon GVCFtyper algorithm.

The subsequent variant call format (VCF) file was used for pF_ST_ analysis using the GPAT++ toolkit within the VCFLIB software library (https://github.com/vcflib; last accessed July 16, 2021; Garrison et al. 2021). pF_ST_ uses a probabilistic approach to detect differences in allele frequencies between populations using a modified-likelihood ratio test that incorporates genotype likelihood information (Kronenberg 2015; Garrison et al. 2021). For orange versus pearl pF_ST_ analysis, the genomes of 28 orange-eyed birds from were compared with the genomes of 33 pearl-eyed birds. For bull eye versus other color pF_ST_ analysis, the genomes of 18 bull-eyed birds were compared with the genomes of 61 nonbull birds (a mix of orange and pearl). For each analysis, the threshold for genome-wide significance was determined by Bonferroni correction (a threshold of 0.05/total number of SNPs assayed).

### Eye Color Phenotyping

Eye colors of birds in our whole-genome resequencing panel were determined from photographs taken at the time of sampling. Each photograph was independently scored by three individuals. In instances where eye color could not confidently be determined from photographs, those individuals were not included in pF_ST_ analysis. Breeds included in the orange-eyed group: American Show Racer, Archangel, Chinese Owl, Damascene, Dragoon, English Carrier, Feral, Granadino Pouter, Hamburg Sticken, Hungarian Giant House Pigeon, Italian Owl, Mindian Fantail, Modena, Pomeranian Pouter, Rafeno Pouter, Saxon Pouter, and Starling. Breeds included in the pearl-eyed group: Australian Tumbler, Bacska Tumbler, Berlin Long-Faced Tumbler, Berlin Short-Faced Tumbler, Birmingham Roller, Budapest Tumbler, Chinese Owl, Cumulet, Danzig Highflier, English Short-Faced Tumbler, English Trumpeter, Fantail, Feral, Helmet, Long Face Tumbler, Naked Neck, Oriental Roller, Polish Lynx, Russian Tumbler, Saint, Temeschburger Schecken, Turkish Tumbler, Uzbek Tumbler, and Vienna Medium-Faced Tumbler. Breeds included in the bull-eyed group: African Owl, Canario Cropper, Classic Old Frill, Chinese Nasal Tuft, English Trumpeter, Fairy Swallow, Ice Pigeon, Komorner Tumbler, Lahore, Mookee, Old German Owl, Oriental Frill, Scandaroon, and Schmalkaldener Mohrenkopf. Eye colors of 93 Pomeranian Pouter x Scandaroon and 66 Archangel x Capuchin F_2_ birds were recorded based on observation at the time of euthanasia, and live photographs showing eye color were taken for reference.

### Plumage Phenotyping

Following euthanasia, photos were taken of F_2_ plumage including dorsal and ventral views with wings and tail spread, and lateral views with wings folded. We divided the body into 15 different regions for phenotyping: dorsal head, right lateral head, left lateral head, dorsal neck, ventral neck, right lateral neck, left lateral neck, dorsal body, ventral body, dorsal tail, ventral tail, dorsal right wing, dorsal left wing, ventral right wing, and ventral left wing. To score each region, we imported photos into Photoshop v21.1.0x64 (Adobe, San Jose, California) and used the magic wand tool to select only the white feathers within the body region. Following this selection, we saved two separate images: one containing the entire region (both pigmented and white feathers) with the color for the white feathers inverted (hereafter, “whole region image”), and one with the selected white feathers removed and only the pigmented feathers remaining (“pigmented region image”). For each body region, we imported these two images into ImageJ (v1.52a; Schneider et al. 2012) and converted them to grayscale, then used the threshold tool to measure the number of pixels in each image. To calculate the proportion of white feathers for each region, we subtracted the number of pixels in the pigmented region image from the number of pixels in the whole region image, then divided by the number of pixels in the whole region image.

### Genotype by Sequencing

DNA samples from founders of the crosses and their F_2_ progeny were extracted using the Qiagen DNeasy Blood and Tissue kit. Our Genotype by Sequencing approach was adapted from a previously published protocol with minor modifications (Elshire et al. 2011; Domyan et al. 2016). DNA was digested with ApeKI, and size selected for fragments in the 550–650 bp range. Domyan et al. (2016) performed an initial round of genotyping for the Pomeranian Pouter x Scandaroon cross. These libraries were sequenced using 100- or 125-bp paired-end sequencing on the Illumina HiSeq2000 platform at the University of Utah Genomics Core Facility. Genotype by sequencing for the Archangel x Capuchin founders (*n* = 2) and F_2_ offspring (*n* = 98), as well as supplemental sequencing for 20 additional and 17 previously low-coverage Pomeranian Pouter x Scandaroon F_2_s, was performed by the University of Minnesota Genomics Center. New GBS libraries were sequenced on a NovaSeq 1 × 100 SP FlowCell.

### Linkage Map Construction

Genotype by sequencing reads were trimmed using CutAdapt (Martin 2011), then mapped to the Cliv_2.1 reference genome reads using Bowtie2 (Langmead and Salzberg 2012). Genotypes were called using Stacks2 by running “refmap.pl” (Catchen et al. 2013). In the Pomeranian Pouter x Scandaroon cross, which had three founders, the Pomeranian Pouter and one of the two Scandaroons designated as parents; to account for the three-founder cross structure, all markers where the genotypes of the two Scandaroon founders differed were subsequently removed from the data set.

We constructed genetic maps using R/qtl v1.46-2 (www.rqtl.org; Broman et al. 2003). Autosomal markers showing significant segregation distortion (*P *<* *0.01 divided by the total number of markers genotyped, to correct for multiple testing) were eliminated. Sex-linked scaffolds were assembled and ordered separately, due to differences in segregation pattern for the Z chromosome. Z-linked scaffolds were identified by assessing sequence similarity and gene content between pigeon scaffolds and the Z chromosome of the annotated chicken genome (Ensembl Gallus_gallus-5.0).

Pairwise recombination frequencies were calculated for all autosomal and Z-linked markers. Markers with identical genotyping information were identified using the “findDupMarkers” command, and all but one marker in each set of duplicates was removed. Within individual Cliv_2.1 scaffolds, markers were filtered by genotyping rate; to retain the maximal number of scaffolds in the final map, an initial round of filtering was performed to remove markers where fewer than 50% of birds were genotyped. Large scaffolds (>40 markers) were subsequently filtered a second time to remove markers where fewer than 66% of birds were genotyped.

Within individual scaffolds, R/Qtl functions “droponemarker” and “calc.errorlod” were used to assess genotyping error. Markers were removed if dropping the marker led to an increased LOD score, or if removing a nonterminal marker led to a decrease in length of >10 cM that was not supported by physical distance. Individual genotypes were removed if they had error LOD scores >5 (a measure of the probability of genotyping error, see Lincoln and Lander [1992]). Linkage groups were assembled from both autosomal markers and Z-linked markers using the parameters (max.rf 0.15, min.lod 6). Scaffolds in the same linkage group were manually ordered based on calculated recombination fractions and LOD scores. Linkage groups in the Pomeranian Pouter x Scandaroon map were numbered by marker number. Linkage groups in the Archangel x Old Dutch Capuchin map were numbered based on scaffold content to correspond with Pomeranian Pouter x Scandaroon linkage groups.

### Quantitative Trait Locus Mapping

We performed QTL mapping using R/qtl v1.46-2 (Broman et al. 2003). For eye color phenotypes, we used the *scanone* function to perform a single-QTL genome scan using a binary model. In QTL scans for the bull eye phenotype, “odd-eyed” birds with a single bull eye were scored as bull. For piebalding phenotypes, we used the *scanone* function to perform a single-QTL genome scan using Haley–Knott regression. For each phenotype, the 5% genome-wide significance threshold was calculated by running the same *scanone* with 1000 permutation replicates. For each significant QTL peak, we calculated 2-LOD support intervals using the *lodint* function. We calculated percent variance explained (PVE) using the *fitqtl* function.

For additional analysis of bull eye QTLs, we again used the *scanone* function to perform a single-QTL scan using a binary model, conditioned on the genotype of the peak QTL marker from the original *scanone* analysis. We assessed both additive and interactive models, and again calculated a significance threshold for each using 1000 permutation replicates.

### Identification of Evolutionarily Conserved Noncoding Regions

We identified four nonavian species (*Terrapene carolina triungis*, three-toed box turtle; *Crocodylus porosus*, saltwater crocodile; *Alligator mississippiensis*, American alligator; *Chrysemys picta bellii*, painted turtle), with syntenic regions spanning the genes *CCDC117*, *SLC2A11B*, *CHEK2*, and *TTC28* using NCBI ortholog annotations and genome browser records. This region contains the pearl eye haplotype identified by pF_ST_. We pulled FASTA sequence from the syntenic region from the NCBI genome browser for each species and aligned these sequencing using mVISTA (Frazer et al. 2004). We defined a highly conserved noncoding region as any region at least 100 bp in length with >70% sequence identity in three or more species that does not overlap annotated exons.

### *SLC2A11B* Mutation Identification and Gene Re-Annotation

We identified numerous SNPs with maximal pF_ST_ scores, and manually examined genotype calls from the VCF file to identify SNPs that followed the expected recessive inheritance pattern of pearl eye (i.e., all pearl-eyed birds were homozygous for the reference allele and all orange-eyed birds were either heterozygous or homozygous for the alternate allele). We identified SLC2A11B orthologs across species using NCBI BLASTp (Altschul et al. 1990; Johnson et al. 2008). The first 10–20 amino acids of the SLC2A11B protein vary across species, but alignments showed that the annotated pigeon protein was missing >80 amino acids that are well conserved most other species, and was likely incomplete. We then took RNA sequences for the orange and pearl alleles of *SLC2A11B* and translated each using Expasy Translate (https://web.expasy.org/translate/; last accessed May 06, 2020; Gasteiger et al. 2003). The longest contiguous protein predicted for the pearl allele matched the protein sequence available on NCBI, whereas the longest contiguous protein for the orange allele was in the same open reading frame, but contained an additional 95 amino acids at the start of the protein sequence. This N-terminal sequence matched the highly conserved SLC2A11B protein sequence annotations across species. The amino acid residue position of the pearl allele mutation is based on this re-annotation.

### Expression Analysis from RNA-Seq Data

RNA-seq data for whole embryos and adult tissues (retina, liver, olfactory epithelium) were obtained from previously described data sets deposited in the SRA database with sequence accessions SRR5878849-SRR5878856 (Holt et al. 2018). For HH25 Oriental Frill and Racing Homer embryo heads, RNA from whole embryonic heads was isolated using the Qiagen RNEasy Kit, and submitted to the University of Utah High Throughput Genomics Shared Resource for Illumina TruSeq stranded library preparation. Libraries were sequenced on the Illumina HiSeq platform. Data are available in NCBI Sequence Read Archive under BioProject PRJNA680754.

We mapped reads to the Cliv_2.1 reference genome using STAR (Dobin et al. 2013), and counted reads in features using FeatureCounts (Liao et al. 2014). Reads per million for the *SLC2A11B* gene and genes within the linkage group 15 QTL were calculated based on total number of uniquely mapped reads per sample. For analysis of *SLC2A11B* expression, we genotyped each sample by looking at reads overlapping two coding SNPs within the pearl eye haplotype (ScoHet5_1307:1895834 and ScoHet5_1307:1896042). We evaluated relative expression level of *SLC2A11B* between orange and pearl alleles using a two-tailed *t*-test to compare reads per million in each sample set. For genes within the linkage group 15 QTL, we grouped samples based on parental and expected eye phenotype: Oriental Frill samples are expected to have bull eyes, as breeding pairs consisted of two piebald bull-eyed birds, whereas Racing Homers are expected to have nonbull eyes (orange or pearl). For each gene, we calculated reads per million for all 12 samples, and performed a *t*-test to identify significant differences in expression. We then took the average reads per million for six Homer samples and six Oriental Frill samples. Log2-fold change was calculated as Log2(Average Homer RPM/Average Oriental Frill RPM).

### Protein Conservation, Structure Prediction, and Mutation Evaluation

We obtained protein sequences for SLC2A11B orthologues across species using NCBI BLASTp and generated multispecies alignments using Clustal Omega (Madeira et al. 2019), and then visualized using Jalview2 (Waterhouse et al. 2009). We assessed the predicted structure of the SLC2A11B protein by using Phobius (Käll et al. 2004) to predict cytoplasmic, noncytoplasmic, transmembrane, and signal peptide domains. As the premature stop codon in *SLC2A11B* occurs very early in the protein sequence, we evaluated the likely impact of the premature stop codon by identifying the next in-frame methionine where translation could initiate to make the longest possible partial protein. We input this truncation into PROVEAN (Choi et al. 2012; Choi and Chan 2015) as a deletion of the first 95 amino acids.

### Gene Ontology Analysis

We mapped gene ontology annotations to identifiers for genes within the two bull eye candidate regions using DAVID v6.8 (https://david.ncifcrf.gov/; last accessed February 25, 2021;Huang et al. 2009). We used annotations from Biological Process (GOTERM_BP_ALL; GOTERM_BP_DIRECT), Cellular Component (GOTERM_CC_ALL; GOTERM_CC_DIRECT), and Molecular Function (GOTERM_MF_ALL; GOTERM_MF_DIRECT) gene ontology databases, and searched results for GO terms containing the keywords “pigment,” “melanosome,” or “melanocyte.”

## Data Availability

Whole-genome sequencing and RNA-seq data sets generated for this study have been deposited to the NCBI SRA database under BioProject PRJNA680754. Previously generated whole-genome sequencing and RNA-seq data used in this study are available under BioProject accessions PRJNA513877, PRJNA428271, and PRJNA167554.

## Supplementary Material

Supplementary data are available at *Molecular Biology and Evolution* online.

## Supplementary Material

msab260_Supplementary_Data

## References

[msab260-B1] Altschul SF , Gish W, Miller W, Myers EW, Lipman DJ. 1990. Basic local alignment search tool. J Mol Biol. 215(3):403–410. 2231712 10.1016/S0022-2836(05)80360-2

[msab260-B2] Amat F , Wollenberg KC, Vences M. 2013. Correlates of eye colour and pattern in mantellid frogs. Salamandra 49:7–17.

[msab260-B3] Andrade P , Gazda MA, Araújo PM, Afonso S, Rasmussen JA, Marques CI, Lopes RJ, Gilbert MTP, Carneiro M. 2021. Molecular parallelisms between pigmentation in the avian iris and the integument of ectothermic vertebrates. PLoS Genet. 17(2):e1009404. 33621224 10.1371/journal.pgen.1009404 PMC7935293

[msab260-B4] Auwera GA , Carneiro MO, Hartl C, Poplin R, Angel G. D, Levy‐Moonshine A, Jordan T, Shakir K, Roazen D, Thibault J, et al 2013. From FastQ data to high‐confidence variant calls: the genome analysis toolkit best practices pipeline. Curr Protoc Bioinform. 43:11.10.1–11.10.33. 10.1002/0471250953.bi1110s43 PMC4243306 25431634

[msab260-B5] Bond C. 1919. On certain factors concerned in the production of eye colour in birds. J Gen. 9(1):69–81.

[msab260-B6] Braasch I , Brunet F, Volff J-N, Schartl M. 2009. Pigmentation pathway evolution after whole-genome duplication in fish. Genome Biol Evol. 1:479–493. 20333216 10.1093/gbe/evp050 PMC2839281

[msab260-B7] Braasch I , Volff J-N, Schartl M. 2009. The endothelin system: evolution of vertebrate-specific ligand-receptor interactions by three rounds of genome duplication. Mol Biol Evol. 26(4):783–799. 19174480 10.1093/molbev/msp015

[msab260-B8] Broman KW , Wu H, Sen Ś, Churchill GA. 2003. R/qtl: QTL mapping in experimental crosses. Bioinformatics 19(7):889–890. 12724300 10.1093/bioinformatics/btg112

[msab260-B9] Bruders R , Hollebeke HV, Osborne EJ, Kronenberg Z, Maclary E, Yandell M, Shapiro MD. 2020. A copy number variant is associated with a spectrum of pigmentation patterns in the rock pigeon (*Columba livia*). PLoS Genet. 16(5):e1008274. 32433666 10.1371/journal.pgen.1008274 PMC7239393

[msab260-B10] Catchen J , Hohenlohe PA, Bassham S, Amores A, Cresko WA. 2013. Stacks: an analysis tool set for population genomics. Mol Ecol. 22(11):3124–3140. 23701397 10.1111/mec.12354 PMC3936987

[msab260-B11] Choi Y , Chan AP. 2015. PROVEAN web server: a tool to predict the functional effect of amino acid substitutions and indels. Bioinformatics 31(16):2745–2747. 25851949 10.1093/bioinformatics/btv195 PMC4528627

[msab260-B12] Choi Y , Sims GE, Murphy S, Miller JR, Chan AP. 2012. Predicting the functional effect of amino acid substitutions and indels. PLoS One. 7(10):e46688. 23056405 10.1371/journal.pone.0046688 PMC3466303

[msab260-B13] Davidson GL , Clayton NS, Thornton A. 2014. Salient eyes deter conspecific nest intruders in wild jackdaws (*Corvus monedula*). Biol Lett. 10(2):20131077. 24501271 10.1098/rsbl.2013.1077 PMC3949376

[msab260-B14] Davidson GL , Thornton A, Clayton NS. 2017. Evolution of iris colour in relation to cavity nesting and parental care in passerine birds. Biol Lett. 13:20160783. 28077686 10.1098/rsbl.2016.0783 PMC5310583

[msab260-B15] Davis-Silberman N , Ashery-Padan R. 2008. Iris development in vertebrates; genetic and molecular considerations. Brain Res. 1192:17–28. 17466284 10.1016/j.brainres.2007.03.043

[msab260-B16] Dobin A , Davis CA, Schlesinger F, Drenkow J, Zaleski C, Jha S, Batut P, Chaisson M, Gingeras TR. 2013. STAR: ultrafast universal RNA-seq aligner. Bioinformatics 29(1):15–21. 23104886 10.1093/bioinformatics/bts635 PMC3530905

[msab260-B17] Doege H , Bocianski A, Scheepers A, Axer H, Eckel J, Joost H-G, Schürmann A. 2001. Characterization of human glucose transporter (GLUT) 11 (encoded by SLC2A11), a novel sugar-transport facilitator specifically expressed in heart and skeletal muscle. Biochem J. 359(Pt 2):443–449. 11583593 10.1042/0264-6021:3590443 PMC1222165

[msab260-B18] Domyan ET , Guernsey MW, Kronenberg Z, Krishnan S, Boissy RE, Vickrey AI, Rodgers C, Cassidy P, Leachman SA, Fondon JW, et al 2014. Epistatic and combinatorial effects of pigmentary gene mutations in the domestic pigeon. Curr Biol. 24(4):459–464. 24508169 10.1016/j.cub.2014.01.020 PMC3990261

[msab260-B19] Domyan ET , Kronenberg Z, Infante CR, Vickrey AI, Stringham SA, Bruders R, Guernsey MW, Park S, Payne J, Beckstead RB, et al 2016. Molecular shifts in limb identity underlie development of feathered feet in two domestic avian species. Elife 5:e12115. 26977633 10.7554/eLife.12115 PMC4805547

[msab260-B20] Edwards M , Cha D, Krithika S, Johnson M, Cook G, Parra EJ. 2016. Iris pigmentation as a quantitative trait: variation in populations of European, East Asian and South Asian ancestry and association with candidate gene polymorphisms. Pigment Cell Melanoma Res. 29(2):141–162. 26547379 10.1111/pcmr.12435

[msab260-B21] Elshire RJ , Glaubitz JC, Sun Q, Poland JA, Kawamoto K, Buckler ES, Mitchell SE. 2011. A robust, simple genotyping-by-sequencing (GBS) approach for high diversity species. PLoS One. 6(5):e19379. 21573248 10.1371/journal.pone.0019379 PMC3087801

[msab260-B22] Frazer KA , Pachter L, Poliakov A, Rubin EM, Dubchak I. 2004. VISTA: computational tools for comparative genomics. Nucleic Acids Res. 32(Web Server issue):W273–W279. 15215394 10.1093/nar/gkh458 PMC441596

[msab260-B23] Gage PJ , Rhoades W, Prucka SK, Hjalt T. 2005. Fate maps of neural crest and mesoderm in the mammalian eye. Invest Ophthalmol Vis Sci. 46(11):4200–4208. 16249499 10.1167/iovs.05-0691

[msab260-B24] Garrison E , Kronenberg ZN, Dawson ET, Pedersen BS, Prins P. 2021. Vcflib and tools for processing the VCF variant call format. bioRxiv: Available from: https://doi.org/10.1101/2021.05.21.445151. 10.1371/journal.pcbi.1009123 PMC9286226 35639788

[msab260-B25] Gasteiger E , Gattiker A, Hoogland C, Ivanyi I, Appel RD, Bairoch A. 2003. ExPASy: the proteomics server for in-depth protein knowledge and analysis. Nucleic Acids Res. 31(13):3784–3788. 12824418 10.1093/nar/gkg563 PMC168970

[msab260-B26] Hamburger V , Hamilton HL. 1951. A series of normal stages in the development of the chick embryo. J Morphol. 88(1):49–92. 24539719

[msab260-B27] Harris ML , Hall R, Erickson CA. 2008. Directing pathfinding along the dorsolateral path - the role of EDNRB2 and EphB2 in overcoming inhibition. Development 135(24):4113–4122. 19004859 10.1242/dev.023119

[msab260-B28] Hoekstra HE. 2006. Genetics, development and evolution of adaptive pigmentation in vertebrates. Heredity 97(3):222–234. 16823403 10.1038/sj.hdy.6800861

[msab260-B29] Hollander WF , Owen RD. 1939. Iris pigmentation in domestic pigeons. Genetica 21(5–6):408–419.

[msab260-B30] Holt C , Campbell M, Keays DA, Edelman N, Kapusta A, Maclary E, Domyan ET, Suh A, Warren WC, Yandell M, et al 2018. Improved genome assembly and annotation for the rock pigeon (*Columba livia*). G3 8(5):1391–1398. 29519939 10.1534/g3.117.300443 PMC5940132

[msab260-B31] Huang DW , Sherman BT, Lempicki RA. 2009. Systematic and integrative analysis of large gene lists using DAVID bioinformatics resources. Nat Protoc. 4(1):44–57. 19131956 10.1038/nprot.2008.211

[msab260-B32] Hubbard JK , Uy JAC, Hauber ME, Hoekstra HE, Safran RJ. 2010. Vertebrate pigmentation: from underlying genes to adaptive function. Trends Genet. 26(5):231–239. 20381892 10.1016/j.tig.2010.02.002

[msab260-B33] Inaba M , Chuong C-M. 2020. Avian pigment pattern formation: developmental control of macro- (across the body) and micro- (within a feather) level of pigment patterns. Front Cell Dev Biol. 8:620. 32754601 10.3389/fcell.2020.00620 PMC7365947

[msab260-B34] Issa S , Bondurand N, Faubert E, Poisson S, Lecerf L, Nitschke P, Deggouj N, Loundon N, Jonard L, David A, et al 2017. EDNRB mutations cause Waardenburg syndrome type II in the heterozygous state. Hum Mutat. 38(5):581–593. 28236341 10.1002/humu.23206

[msab260-B35] Jabeen R , Babar ME, Ahmad J, Awan AR. 2012. Novel mutations of endothelin-B receptor gene in Pakistani patients with Waardenburg syndrome. Mol Biol Rep. 39(1):785–788. 21547364 10.1007/s11033-011-0799-x

[msab260-B36] Johnson M , Zaretskaya I, Raytselis Y, Merezhuk Y, McGinnis S, Madden TL. 2008. NCBI BLAST: a better web interface. Nucleic Acids Res. 36(Web Server issue):W5–W9. 18440982 10.1093/nar/gkn201 PMC2447716

[msab260-B37] Kaelin CB , Barsh GS. 2013. Genetics of pigmentation in dogs and cats. Annu Rev Anim Biosci. 1:125–156. 25387014 10.1146/annurev-animal-031412-103659

[msab260-B38] Käll L , Krogh A, Sonnhammer ELL. 2004. A combined transmembrane topology and signal peptide prediction method. J Mol Biol. 338(5):1027–1036. 15111065 10.1016/j.jmb.2004.03.016

[msab260-B39] Kelsh RN , Harris ML, Colanesi S, Erickson CA. 2009. Stripes and belly-spots – a review of pigment cell morphogenesis in vertebrates. Semin Cell Dev Biol. 20(1):90–104. 18977309 10.1016/j.semcdb.2008.10.001 PMC2744437

[msab260-B40] Kimura T , Nagao Y, Hashimoto H, Yamamoto-Shiraishi Y, Yamamoto S, Yabe T, Takada S, Kinoshita M, Kuroiwa A, Naruse K. 2014. Leucophores are similar to xanthophores in their specification and differentiation processes in medaka. Proc Natl Acad Sci U S A. 111(20):7343–7348. 24803434 10.1073/pnas.1311254111 PMC4034200

[msab260-B41] Kinoshita K , Akiyama T, Mizutani M, Shinomiya A, Ishikawa A, Younis HH, Tsudzuki M, Namikawa T, Matsuda Y. 2014. Endothelin receptor B2 (EDNRB2) is responsible for the tyrosinase-independent recessive white (mo(w)) and mottled (mo) plumage phenotypes in the chicken. PLoS One. 9(1):e86361. 24466053 10.1371/journal.pone.0086361 PMC3900529

[msab260-B42] Koide T , Moriwaki K, Uchida K, Mita A, Sagai T, Yonekawa H, Katoh H, Miyashita N, Tsuchiya K, Nielsen TJ, et al 1998. A new inbred strain JF1 established from Japanese fancy mouse carrying the classic piebald allele. Mamm Genome. 9(1):15–19. 9434939 10.1007/s003359900672

[msab260-B43] Kronenberg Z. 2015. Genotype-phenotype association using high throughput sequencing data [doctoral dissertation]. Salt Lake City (UT): J. Willard Marriott Digital Library, University of Utah. https://collections.lib.utah.edu/ark:/87278/s6dg18z9. Accessed July 16, 2021.

[msab260-B44] Langmead B , Salzberg SL. 2012. Fast gapped-read alignment with Bowtie 2. Nat Methods. 9(4):357–359. 22388286 10.1038/nmeth.1923 PMC3322381

[msab260-B45] Levi W. 1986. The Pigeon. 2nd rev. ed. Sumter [SC]: Levi Publishing Co., Inc.

[msab260-B46] Li L , Li D, Liu L, Li S, Feng Y, Peng X, Gong Y. 2015. Endothelin receptor B2 (EDNRB2) gene is associated with spot plumage pattern in domestic ducks (*Anas platyrhynchos*). PLoS One. 10(5):e0125883. 25955279 10.1371/journal.pone.0125883 PMC4425580

[msab260-B47] Liao Y , Smyth GK, Shi W. 2014. featureCounts: an efficient general purpose program for assigning sequence reads to genomic features. Bioinformatics 30(7):923–930. 24227677 10.1093/bioinformatics/btt656

[msab260-B48] Lincoln SE , Lander ES. 1992. Systematic detection of errors in genetic linkage data. Genomics 14(3):604–610. 1427888 10.1016/s0888-7543(05)80158-2

[msab260-B49] Linnen CR , Poh Y-P, Peterson BK, Barrett RDH, Larson JG, Jensen JD, Hoekstra HE. 2013. Adaptive evolution of multiple traits through multiple mutations at a single gene. Science 339(6125):1312–1316. 23493712 10.1126/science.1233213 PMC3836219

[msab260-B50] Liu XY , Dangel AW, Kelley RI, Zhao W, Denny P, Botcherby M, Cattanach B, Peters J, Hunsicker PR, Mallon A-M, et al 1999. The gene mutated in bare patches and striated mice encodes a novel 3β-hydroxysteroid dehydrogenase. Nat Genet. 22(2):182–187. 10369263 10.1038/9700

[msab260-B51] Lorin T , Brunet FG, Laudet V, Volff J-N. 2018. Teleost fish-specific preferential retention of pigmentation gene-containing families after whole genome duplications in vertebrates. G3 8(5):1795–1806. 29599177 10.1534/g3.118.200201 PMC5940169

[msab260-B52] Madeira F , Park Y. M, Lee J, Buso N, Gur T, Madhusoodanan N, Basutkar P, Tivey ARN, Potter SC, Finn RD, et al 2019. The EMBL-EBI search and sequence analysis tools APIs in 2019. Nucleic Acids Res. 47(W1):W636–W641. 30976793 10.1093/nar/gkz268 PMC6602479

[msab260-B53] Madge S. 2020. Hooded Crow (*Corvus cornix*), version 1.0. In: Billerman SM, Keeney BK, Rodewald PG, Schulenberg TS, editors. Birds of the World. Ithaca [NY]: Cornell Lab of Ornithology. 10.2173/bow.hoocro1.01.

[msab260-B54] Martin M. 2011. Cutadapt removes adapter sequences from high-throughput sequencing reads. Embnet J. 17(1):10–12.

[msab260-B55] Metallinos DL , Bowling AT, Rine J. 1998. A missense mutation in the endothelin-B receptor gene is associated with Lethal White Foal Syndrome: an equine version of Hirschsprung disease. Mamm Genome. 9(6):426–431. 9585428 10.1007/s003359900790

[msab260-B56] Miwa M , Inoue-Murayama M, Aoki H, Kunisada T, Hiragaki T, Mizutani M, Ito S. 2007. Endothelin receptor B2 (EDNRB2) is associated with the panda plumage colour mutation in Japanese quail: quail plumage colour mutation associated with EDNRB2. Anim Genet. 38(2):103–108. 17313575 10.1111/j.1365-2052.2007.01568.x

[msab260-B57] Moscatelli G , Bovo S, Schiavo G, Mazzoni G, Bertolini F, Dall'Olio S, Fontanesi L. 2020. Genome‐wide association studies for iris pigmentation and heterochromia patterns in Large White pigs. Anim Genet. 51(3):409–419. 32232994 10.1111/age.12930

[msab260-B58] Negro JJ , Blázquez MC, Galván I. 2017. Intraspecific eye color variability in birds and mammals: a recent evolutionary event exclusive to humans and domestic animals. Front Zool. 14:53. 29225658 10.1186/s12983-017-0243-8 PMC5716259

[msab260-B59] Oliphant LW. 1987a. Pteridines and purines as major pigments of the avian iris. Pigment Cell Res. 1(2):129–131. 3507666 10.1111/j.1600-0749.1987.tb00401.x

[msab260-B60] Oliphant LW. 1987b. Observations on the pigmentation of the pigeon iris. Pigment Cell Res. 1(3):202–208. 3508278 10.1111/j.1600-0749.1987.tb00414.x

[msab260-B61] Parichy DM , Spiewak JE. 2015. Origins of adult pigmentation: diversity in pigment stem cell lineages and implications for pattern evolution. Pigment Cell Melanoma Res. 28(1):31–50. 25421288 10.1111/pcmr.12332 PMC4276524

[msab260-B62] Passarotto A , Parejo D, Cruz‐Miralles A, Avilés JM. 2018. The evolution of iris colour in relation to nocturnality in owls. J Avian Biol. 49(12). doi: 10.1111/jav.01908.

[msab260-B63] Pingault V , Ente D, Moal FD, Goossens M, Marlin S, Bondurand N. 2010. Review and update of mutations causing Waardenburg syndrome. Hum Mutat. 31(4):391–406. 20127975 10.1002/humu.21211

[msab260-B64] Pla P , Alberti C, Solov'eva O, Pasdar M, Kunisada T, Larue L. 2005. Ednrb2 orients cell migration towards the dorsolateral neural crest pathway and promotes melanocyte differentiation. Pigment Cell Res. 18(3):181–187. 15892714 10.1111/j.1600-0749.2005.00230.x

[msab260-B65] Read AP , Newton VE. 1997. Waardenburg syndrome. J Med Genet. 34(8):656–665. 9279758 10.1136/jmg.34.8.656 PMC1051028

[msab260-B66] Sanchez-Ferras O , Bernas G, Farnos O, Touré AM, Souchkova O, Pilon N. 2016. A direct role for murine Cdx proteins in the trunk neural crest gene regulatory network. Development. 143(8):1363–1374. 26952979 10.1242/dev.132159

[msab260-B67] Schneider CA , Rasband WS, Eliceiri KW. 2012. NIH image to ImageJ: 25 years of image analysis. Nat Methods. 9(7):671–675. 22930834 10.1038/nmeth.2089 PMC5554542

[msab260-B68] Scott GA , Jacobs SE, Pentland AP. 2006. sPLA2-X stimulates cutaneous melanocyte dendricity and pigmentation through a lysophosphatidylcholine-dependent mechanism. J Invest Dermatol. 126(4):855–861. 16456529 10.1038/sj.jid.5700180

[msab260-B69] Sell A. 2012. Pigeon Genetics: Applied Genetics in the Domestic Pigeon. Achim, Germany: Sell Publishing.

[msab260-B70] Shapiro MD , Kronenberg Z, Li C, Domyan ET, Pan H, Campbell M, Tan H, Huff CD, Hu H, Vickrey AI, et al 2013. Genomic diversity and evolution of the head crest in the rock pigeon. Science 339(6123):1063–1067. 23371554 10.1126/science.1230422 PMC3778192

[msab260-B71] Si S , Xu X, Zhuang Y, Gao X, Zhang H, Zou Z, Luo S-J. 2021. The genetics and evolution of eye color in domestic pigeons (*Columba livia*). *PLoS Genet*. 17(8):e1009770. 10.1371/journal.pgen.1009770 PMC8432899 34460822

[msab260-B72] Snow D. 2020. Wire-tailed manakin (*Pipra filicauda*), version 1.0. In: del Hoyo J, Elliott A, Sargatal J, Christie DA, de Juana E, editors. Birds of the World. Ithaca [NY]: Cornell Lab of Ornithology. 10.2173/bow.witman2.01.

[msab260-B73] Square T , Jandzik D, Cattell M, Hansen A, Medeiros DM. 2016. Embryonic expression of endothelins and their receptors in lamprey and frog reveals stem vertebrate origins of complex Endothelin signaling. Sci Rep. 6:34282. 27677704 10.1038/srep34282 PMC5039696

[msab260-B74] Sturm RA , Larsson M. 2009. Genetics of human iris colour and patterns. Pigment Cell Melanoma Res. 22(5):544–562. 19619260 10.1111/j.1755-148X.2009.00606.x

[msab260-B75] Tamura K , Ohbayashi N, Ishibashi K, Fukuda M. 2011. Structure-function analysis of VPS9-ankyrin-repeat protein (Varp) in the trafficking of tyrosinase-related protein 1 in melanocytes. J Biol Chem. 286(9):7507–7521. 21187289 10.1074/jbc.M110.191205 PMC3045006

[msab260-B76] Vickrey AI , Bruders R, Kronenberg Z, Mackey E, Bohlender RJ, Maclary ET, Maynez R, Osborne EJ, Johnson KP, Huff CD, et al. 2018. Introgression of regulatory alleles and a missense coding mutation drive plumage pattern diversity in the rock pigeon. Elife 7:e34803. 30014848 10.7554/eLife.34803 PMC6050045

[msab260-B77] Waterhouse AM , Procter JB, Martin DMA, Clamp M, Barton GJ. 2009. Jalview version 2—a multiple sequence alignment editor and analysis workbench. Bioinformatics 25(9):1189–1191. 19151095 10.1093/bioinformatics/btp033 PMC2672624

[msab260-B78] Wu N , Qin H, Wang M, Bian Y, Dong B, Sun G, Zhao W, Chang G, Xu Q, Chen G. 2017. Variations in endothelin receptor B subtype 2 (EDNRB2) coding sequences and mRNA expression levels in 4 Muscovy duck plumage colour phenotypes. Br Poult Sci. 58(2):116–121. 27879145 10.1080/00071668.2016.1259531

[msab260-B79] Xi Y , Wang L, Liu H, Ma S, Li Y, Li L, Wang J, Chunchun H, Bai L, Mustafa A, et al 2020. A 14-bp insertion in endothelin receptor B-like (EDNRB2) is associated with white plumage in Chinese geese. BMC Genomics. 21(1):162. 32066369 10.1186/s12864-020-6562-8 PMC7027040

[msab260-B80] Zdobnov EM , Apweiler R. 2001. InterProScan – an integration platform for the signature-recognition methods in InterPro. Bioinformatics 17(9):847–848. 11590104 10.1093/bioinformatics/17.9.847

