# Supplementary material for: Two Genomic Loci Control Three Eye Colors in the Domestic Pigeon (Columba livia)
Source: Mol Biol Evol. 2021 Aug 30;38(12):5376–90. doi: 10.1093/molbev/msab260 (PMC8662629; doi:10.1093/molbev/msab260)
Supplement: msab260_Supplementary_Data [file msab260_supplementary_data.pdf]

Supplementary Information: Two Genomic Loci Control Three Eye Colors in the Domestic Pigeon (*Columba livia*)

Authors: Emily T. Maclary[1], Bridget Phillips[1], Ryan Wauer[1], Elena F. Boer[1], Rebecca Bruders[1], Tyler Gilvarry[1], Carson Holt[2], Mark Yandell[2], and Michael D. Shapiro[1]

Affiliations:
[1]School of Biological Sciences, University of Utah, Salt Lake City, UT 84112, USA
[2] Department of Human Genetics and Utah Center for Genetic Discovery, University of Utah, Salt Lake City, UT, USA

*Author for Correspondence:
Michael D. Shapiro, School of Biological Sciences, 257 South 1400 East, Salt Lake City, UT 84112 USA; phone +1 801 581 5690; email: mike.shapiro@utah.edu

Figure S1
Figure S2
Figure S3
Figure S4
Figure S5
Figure S6
Figure S7
Table S1
Table S2

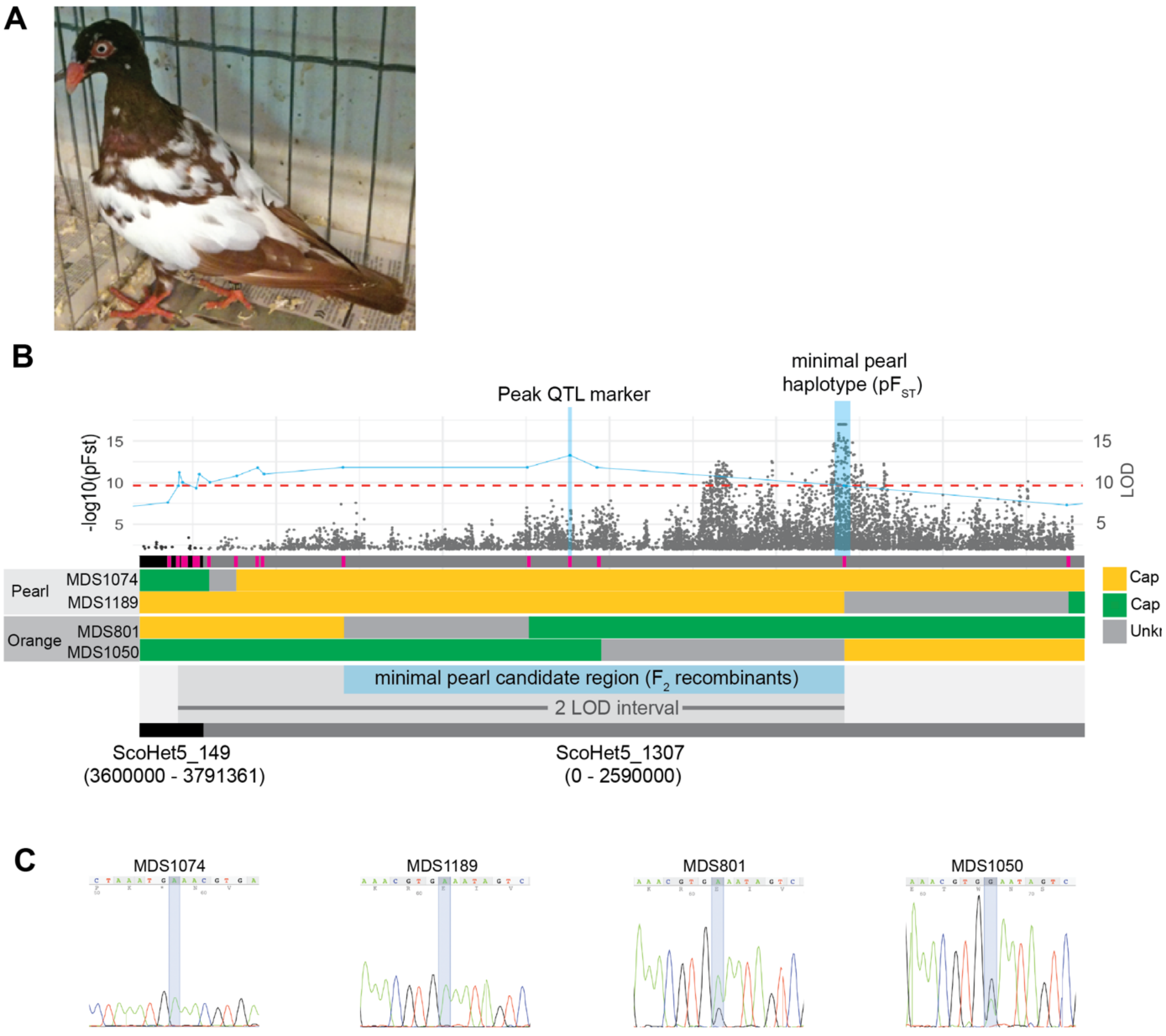

Figure S1. QTL mapping and pF$_{ST}$ identify a single candidate region for pearl eye color. (A) The pigeon sequenced for the *C. livia* reference genome, a Danish Tumbler with pearl eyes [from Shapiro et al. 2013]. (B) Overlay of multi-breed pearl eye pF$_{ST}$ (gray dots, score indicated on left-side Y axis indicates -log10(pF$_{ST}$)) and pearl eye QTL from the Archangel x Capuchin F2 intercross (blue line, right-side Y axis indicates LOD score). The minimal pearl eye haplotype from pF$_{ST}$ and peak QTL marker are both highlighted in blue. X axis indicates position on the significant portion of Linkage Group 20. Full plot spans the last 0.2 mb of scaffold ScoHet5_149 and the first 2.6 mb of scaffold ScoHet5_1307. Pink lines indicate the location of QTL markers. Below, the genotypes of six individual F$_2$ birds from the Archangel x Old Dutch Capuchin cross are plotted. Yellow indicates regions that are homozygous for the Capuchin allele. Green indicates heterozygous regions. Gray regions show areas where a recombination event has occurred between flanking markers. Below recombinants, the 2-LOD QTL interval is indicated in gray and the minimal candidate region as defined by individual recombinants is indicated in blue. The left-side boundary of the minimal candidate region is defined by a recombination event in MDS801; the right-side boundary is defined by recombination events in MDS1050. (C) Chromatograms showing genotyping results for a SNP at ScoHet5_1307: 1895934 in F$_2$ recombinant individuals 1074, 1189, 801, and 1050.

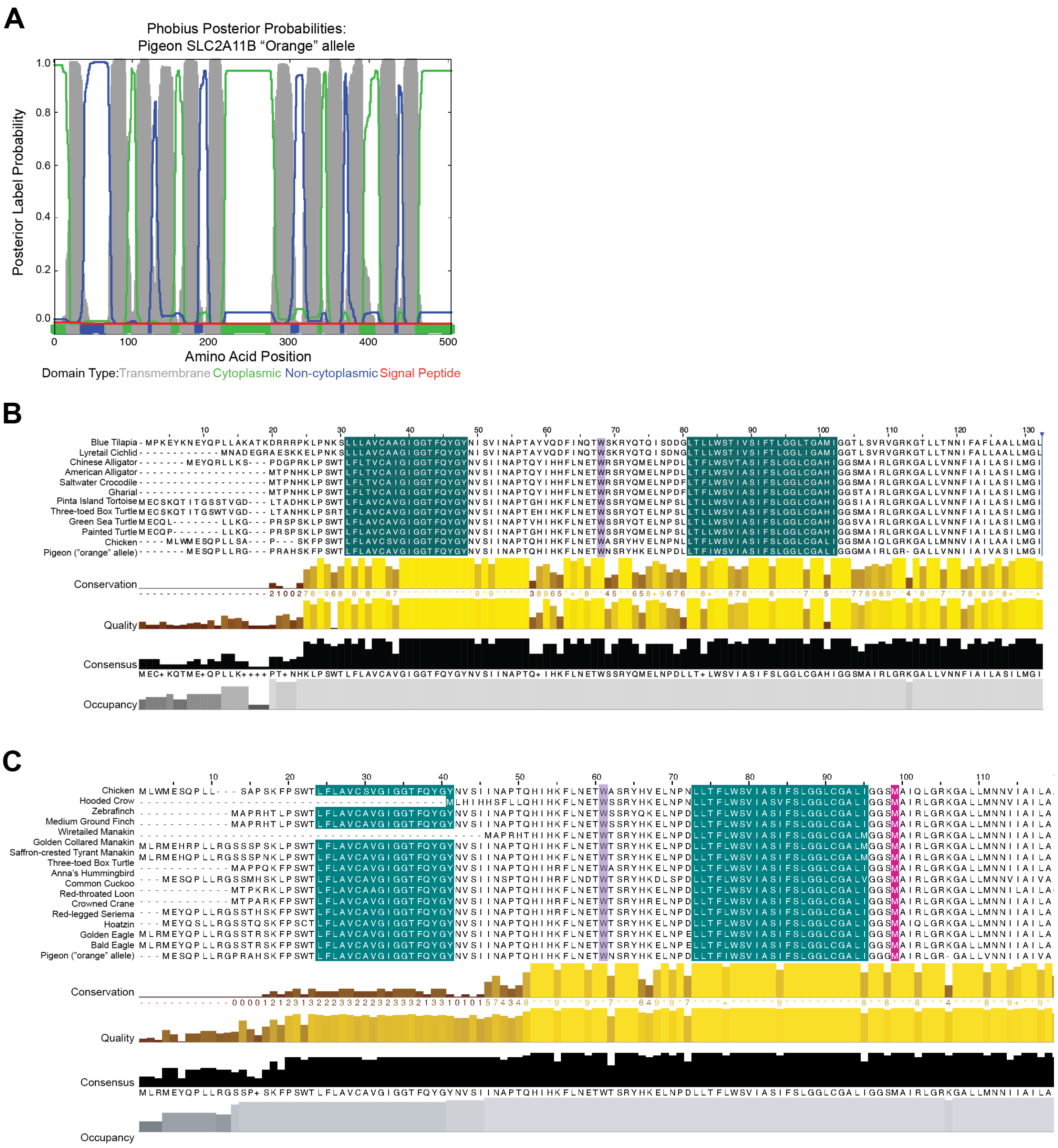

Figure S2. A premature stop codon in exon 3 of *SLC2A11B* is predicted to disrupt conserved protein structure and function. (A) Sequence-based domain prediction of SLC2A11B structure from Phobius. Amino acid positions of protein regions are depicted on the X axis and are color-coded by predicted region type. Probability scores for each predicted domain type are shown on the Y axis. (B) Multi-species alignment of SLC2A11B protein sequence from 12 fish and sauropsid species, including pigeon. Teal color indicates the spans of the first two transmembrane domains predicted by Phobius, which are highly conserved across species. The residue affected by the pearl eye mutation, W58 in the pigeon orange allele protein sequence, is highlighted in purple. (C) Multi-species alignment of SLC2A11B protein sequence from 17 bird species, including pigeon. Teal and purple indicate same features as in (B). Pink indicates the next in-frame methionine after the pearl eye W58X mutation from which translation could initiate.

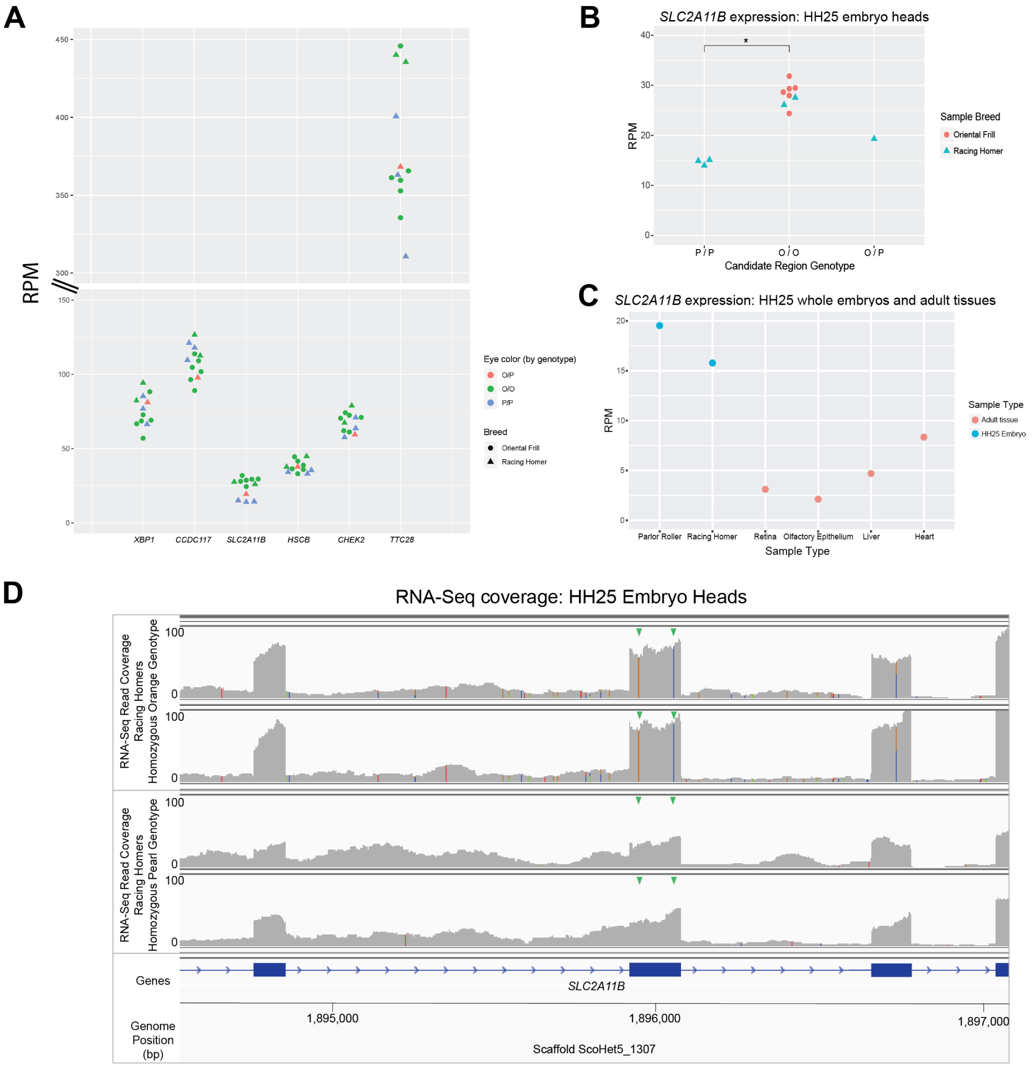

Figure S3. Spliced *SLC2A11B* transcript counts are reduced in embryos carrying the pearl allele. (A) Plot of normalized RNA-seq read counts for all genes within 15 kb of the pearl eye haplotype from whole HH25 embryo heads of two different breeds, Racing Homer and Oriental Frill. RPM, uniquely mapped RNA-seq reads. P/P (n=3), embryos homozygous for the pearl haplotype. O/O (n=8), embryos homozygous for the orange haplotype. O/P (n=1), heterozygous embryo. Only *SLC2A11B* shows stratification in expression based on genotype, which is more apparent in (B). (B) Plot of normalized *SLC2A11B* read count in whole HH25 embryo Racing Homer and Oriental Frill Y axis shows normalized read count. Expression is significantly reduced (p = 1.139e-07, two-tailed t test) in embryos homozygous for the pearl allele (P/P) compared to embryos homozygous for the orange allele (O/O). One heterozygous sample (O/P) shows intermediate expression. (B) Plot of normalized *SLC2A11B* read count in RNA-seq data from whole HH25 embryos (one Parlor Roller, one Racing Homer) and four adult tissues (data from Holt et al. 2018). Y axis shows normalized read count. (C) Visual evaluation of mapped RNA-seq reads shows an apparent increase in the proportion of intronic reads in samples homozygous for the pearl allele. Each track represents a single sample. Y axis shows non-normalized RNA-seq coverage. X axis shows base pair positions across the region, *SLC2A11B* exons are indicated in blue. Green arrowheads point to the locations of two exonic single nucleotide polymorphisms associated with eye color. These SNPs are highlighted in the orange genotype samples (upper two tracks) as the sequence in these samples differs from the pearl-eyed reference genome. Additional colored lines in each sample mark non-reference alleles.

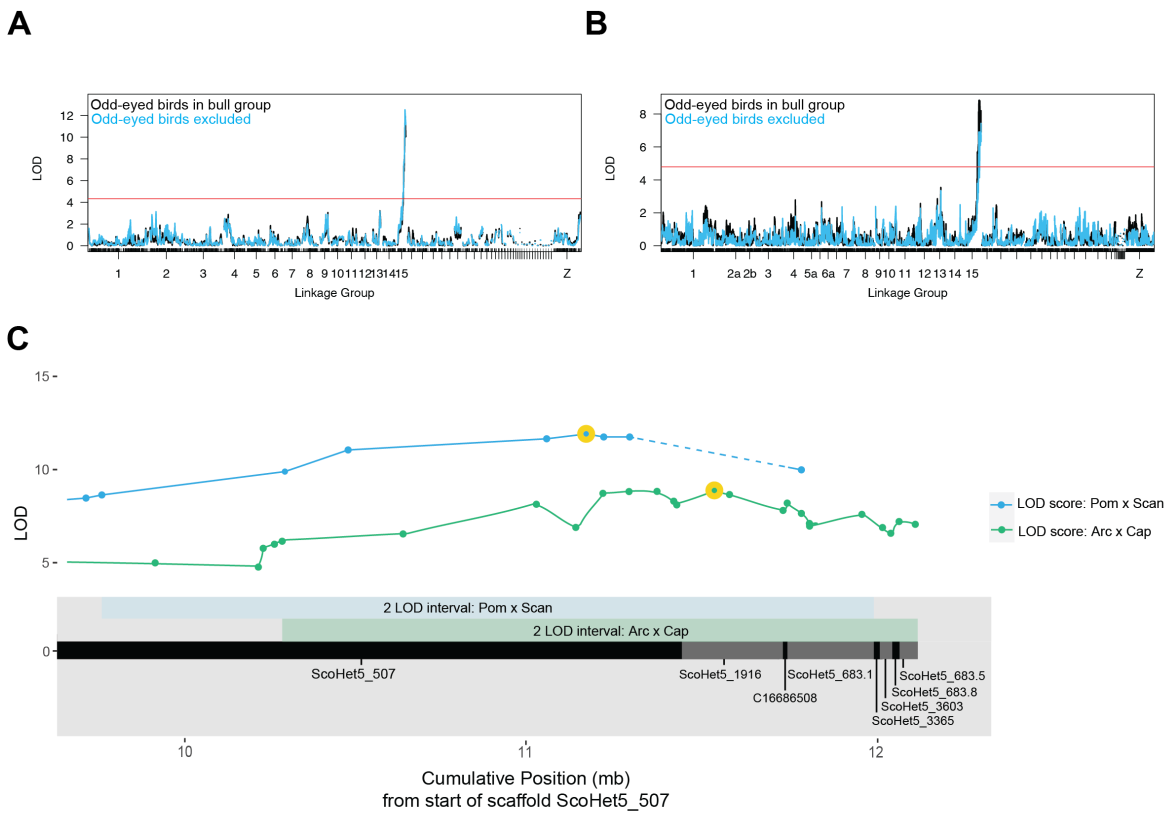

Figure S4. Bull eye QTLs overlap in two crosses. (A) The same LG15 QTL shown in Fig. 2 is identified in the Pomeranian Pouter x Scandaroon cross when odd-eyed birds are excluded from the sample. Black line, original QTL with odd-eyed birds included in the "bull" group, blue line, QTL with odd-eyed birds excluded. Red dashed line indicates genome-wide significance threshold. (B) The same LG15 QTL seen in Fig. 2 is identified in the Archangel x Capuchin cross when odd-eyed birds are excluded from the sample. Black line, original QTL with odd-eyed birds included in the "bull" group, blue line, QTL with odd-eyed birds excluded. Red dashed line indicates genome-wide significance threshold. (C) QTL results for bull eye color in the Pomeranian Pouter x Scandaroon (blue) and Archangel x Capuchin (green) crosses. Peak marker for each cross is highlighted in yellow. 2-LOD intervals are indicated below. The X axis shows cumulative physical position (Mb) on LG15. Black and gray bars indicate scaffold boundaries for the eight scaffolds in the Archangel x Capuchin linkage group 15; six of these scaffolds are not represented in the Pomeranian Pouter x Scandaroon cross due to an absence of informative markers on these scaffolds. The dashed portion of the blue Pomeranian Pouter x Scandaroon line indicates the region spanning scaffolds (ScoHet5_1916 and C16686508) that are absent from this cross. The remaining scaffolds that lack informative markers in the Pomeranian Pouter x Scandaroon cross are at the end of the linkage group.

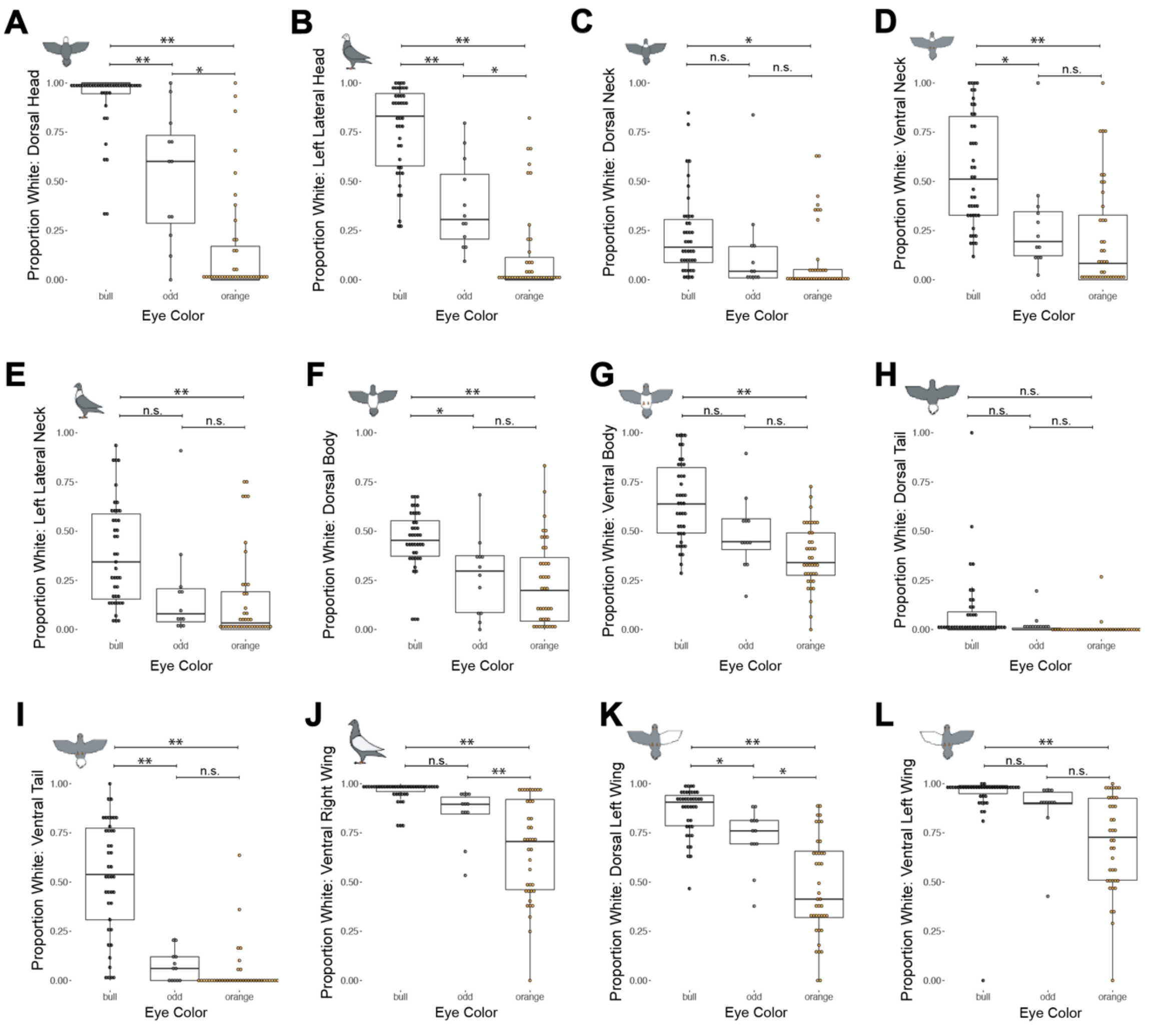

Figure S5. Association between plumage pigmentation and eye color in Pomeranian Pouter x Scandaroon F_2 offspring. (A-L) Boxplots depicting the proportion of white plumage on the indicated body region in Pomeranian Pouter x Scandaroon F_2 birds with bull, odd, or orange eyes. **, $p \leq 0.0001$; *, $0.001 < p \leq 0.01$; n.s., $p > 0.01$. Boxes span from the first to third quartile of each data set, with lines indicating the median. Whiskers span up to 1.5x the interquartile range.

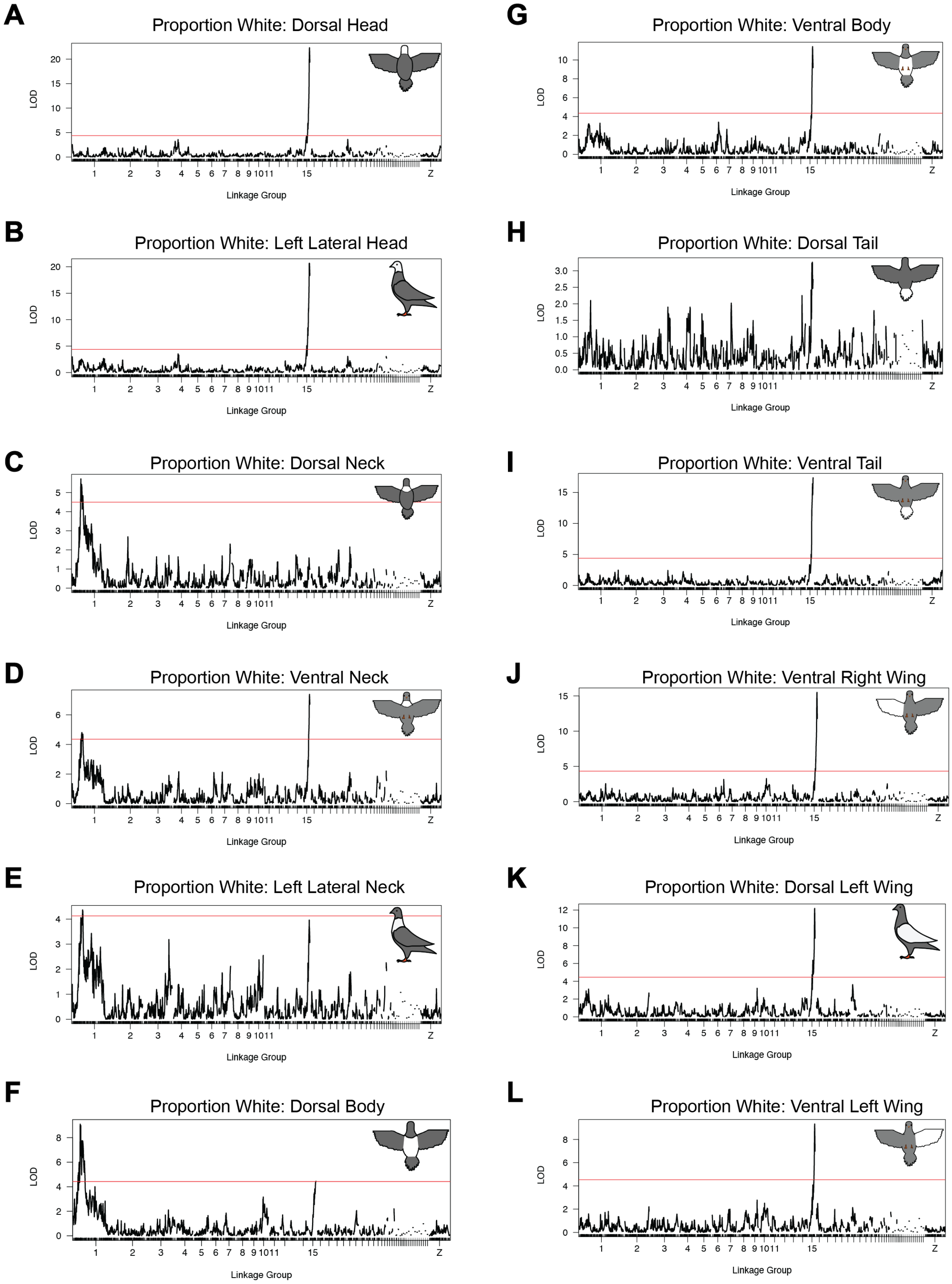

Figure S6. Two linkage groups are associated with regionally-specific white plumage in a Pomeranian Pouter x Scandaroon F_2 intercross. (A-L) Genome-wide QTL scans for proportion of white plumage on the indicated body region. Red lines indicate the 5% genome-wide significance threshold. In (H), a significant QTL was not identified for the dorsal tail region.

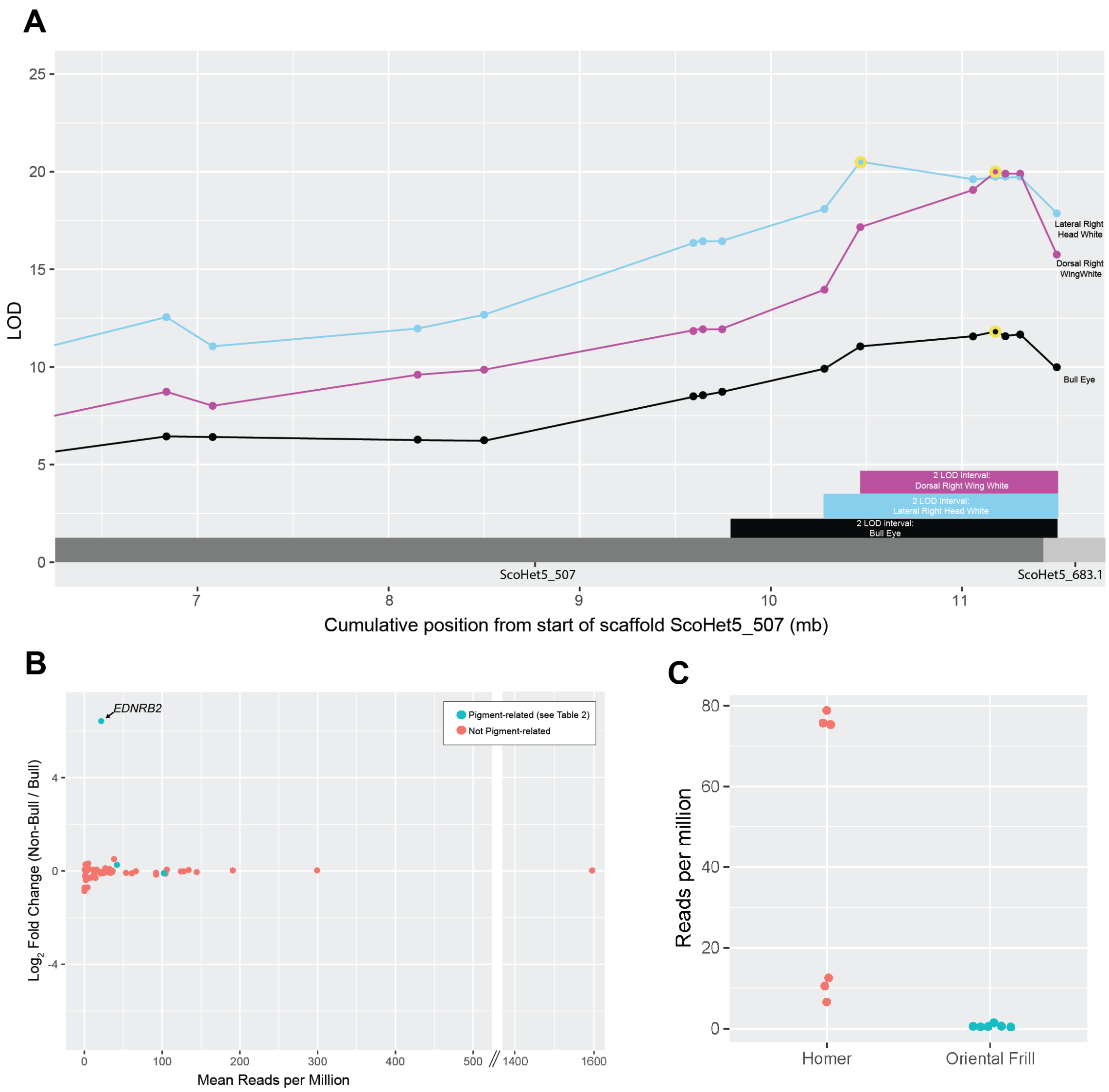

Figure S7. Evaluation of the overlapping bull eye and piebalding QTL regions. (A) Plot of bull eye (black line), dorsal right wing white (pink) and lateral right head white (blue) QTLs from the Pomeranian Pouter x Scandaroon cross. Y axis shows LOD score. X axis shows physical position in Mb. Boxes indicate the 2-LOD intervals for each QTL. (B) Plot of Log2-fold change for genes in the LG15 bull eye QTL region (both Archangel x Capuchin and Pomeranian Pouter x Scandaroon 2-LOD intervals) between Racing Homer (non-bull, non-pied) and Oriental Frill (bull eyed, piebald) HH25 embryo heads. X axis, mean reads per million across all twelve samples from both breeds. Y axis, Log2(Homer RPM/Oriental Frill RPM). *EDNRB2* shows a drastic change in expression between breeds. (C) Plot of reads per million in the *EDNRB2* gene for Homer (left, not pied and non-bull) and Oriental Frill (right, pied and bull eyed).

Table S1: Differentially Expressed Genes in HH25 Homer vs. Oriental Frill Heads

Gene	Position	Reads per Million: Oriental Frills (Pied, bull eye)						Reads per Million: Homers (Non-pied, Non-bull)						Log2 FC (Hom/OF)	P Value (T test)
		OF1	OF2	OF3	OF4	OF5	OF6	Hom1	Hom2	Hom3	Hom4	Hom5	Hom6		
RPAC2	ScoHet5_507: 11146234-11157658	30.63	31.03	30.92	31.68	32.16	32.64	40.02	45.84	53.20	39.82	39.84	50.09	0.51	0.0012
CD244	ScoHet5_507: 11421668-11438299	6.07	4.00	5.93	5.16	5.30	4.32	1.92	2.70	2.66	2.74	5.27	3.57	-0.71	0.0039
CHIC1[1]	ScoHet5_507: 9977959-9994285	5.04	3.79	3.58	4.95	4.66	5.07	5.88	6.01	6.06	4.74	5.91	5.13	0.32	0.0054
RAB33A[2]	ScoHet5_1916: 88553-95797	4.90	5.03	5.12	4.87	6.64	5.14	3.85	3.98	3.34	5.31	4.31	4.61	-0.32	0.0112
VAMP7	ScoHet5_507: 11126494-11144082	101.82	100.60	106.47	104.08	113.48	108.52	97.41	102.10	104.86	98.19	92.59	98.76	-0.10	0.0124
LOC102083831	ScoHet5_507: 10719598-10724486	16.21	17.10	17.16	13.62	18.29	11.82	14.69	11.83	12.50	12.95	11.50	13.11	-0.30	0.0164
EDNRB2	ScoHet5_507: 11162676-11176857	0.34	0.48	0.31	0.50	0.41	0.95	4.06	78.81	75.29	10.50	12.53	75.72	6.42	0.0192
NSDHL	ScoHet5_507: 11023840-11035275	33.32	39.16	34.01	38.21	36.70	47.30	45.63	38.85	49.92	43.87	39.71	56.39	0.26	0.0252

[1]Gene outside of Archangel x Capuchin 2-LOD interval
[2]Gene on scaffold not present in Pomeranian Pouter x Scandaroon linkage map

Table S2. Sample IDs and sequencing coverage for birds included in pF_{ST} analyses.

Sample ID	Breed	Eye Color	Average depth of coverage
10385X14_FER_3087_CN	Feral	Orange	1.36
10385X18_FER_4168_CW	Feral	Orange	1.61
10385X20_ITO_51_B	Italian Owl	Orange	1.33
10385X22_FER_3020_CN	Feral	Orange	1.92
10385X25_CHO_52_B	Chinese Owl	Orange	1.52
10385X2_STA_43_B	Starling	Orange	3.01
10385X30_POM_ZK26_B	Pomeranian Pouter	Orange	1.68
10385X38_FER_4180_BW	Feral	Orange	1.78
10385X39_FER_4151_CW	Feral	Orange	1.82
10385X41_FER_2882_CN	Feral	Orange	2.64
10385X45_FER_4177_CW	Feral	Orange	2.01
10385X4_FER_4152_CW	Feral	Orange	2.86
10385X6_ASR_26_B	American Show Racer	Orange	1.95
10385X7_FER_4035_BN	Feral	Orange	1.58
10385X9_FER_4076_BW	Feral	Orange	1.81
11573X2_MOD_UPS14-26_barless	Modena	Orange	16.33
11573X4_ARC_UPS14-51_barless	Archangel	Orange	15.60
11573X5_CHO_UPS14-55_barless	Chinese Owl	Orange	18.12
12319X5_RAF_UPS15-28_C_M	Rafeno Pouter	Orange	18.48
14064X8_ITO_UPS16_09_RED_M	Italian Owl	Orange	14.53
RBIDIAAPE_SAP_BGI	Saxon Pouter	Orange	25.13
15778X5_HGH_UPS15-86	Hungarian Giant House Pigeon	Orange	20.16
15778X12_HAM_UPS18-16	Hamburg Sticken	Orange	17.02
15778X16_MIF_UPS10-316	Mindian Fantail	Orange	18.11
16238X2_GRA_UPS18-21	Granadino Pouter	Orange	20.86
16238X5_DAM_UPS18-20	Damascene	Orange	19.35
16238X6_DRA_UPS16-20	Dragoon	Orange	17.29
16238X7_ENC_UPS16-11	English Carrier	Orange	18.60
10385X12_FAN_50_B	Fantail	Pearl	1.52
10385X15_BUT_214_C	Budapest Tumbler	Pearl	1.51
10385X16_BUT_309_B	Budapest Tumbler	Pearl	1.34
10385X36_POL_188_C	Polish Lynx	Pearl	2.11
10385X3_FER_4033_BW	Feral	Pearl	2.72
10385X43_FER_4059_CW	Feral	Pearl	2.09
10385X46_FER_2884_BN	Feral	Pearl	2.16
11186X11_BAC_UPS12-092_R_M	Bacska Tumbler	Pearl	14.68
11186X8_CHO_UPS08-41_ALM_F	Chinese Owl	Pearl	5.96
11186X9_ORR_UPS08-140_ALM_F	Oriental Roller	Pearl	6.96
11298X2_UZT_GN68_muff	Uzbek Tumbler	Pearl	18.48
11573X7_BIR_CT15-05_barless	Birmingham Roller	Pearl	15.16
12097X3_ENT_UPS15-61_ALM_F	English Trumpeter	Pearl	17.21
12319X1_EST_UPS15-07_DUN_M	English Short Faced Tumbler	Pearl	19.26
12319X2_BST_UPS15-08_GRZ_M	Berlin Short Faced Tumbler	Pearl	17.41
12319X3_BST_UPS15-15_GRZ_F	Berlin Short Faced Tumbler	Pearl	15.57
14064X3_LFT_UPS16_99_WHI_M	Long Faced Tumbler	Pearl	18.55
14064X5_HEL_GN236_SPR_M	Helmet	Pearl	14.16
14064X7_BUT_GN321_M	Budapest Tumbler	Pearl	14.61

15321X2_BST_UPS08_148_KHA_F	Berlin Short Faced Tumbler	Pearl	19.10
15321X3_BUT_GN216_RED_F	Budapest Tumbler	Pearl	17.67
15321X6_EST_UPS14_35_ALM_M	English Short Faced Tumbler	Pearl	19.75
15321X7_HEL_GN324_RED_M	Helmet	Pearl	20.85
RAZDIAAPE_CUM_BGI	Cumulet	Pearl	18.00
15778X1_DAH_UPS08-92	Danzig Highflier	Pearl	18.95
15778X2_TEM_UPS08-94	Temeschburger Schecken	Pearl	18.74
15778X7_SAI_GN230	Saint	Pearl	19.97
15778X10_RUS_UPS17-07	Russian Tumbler	Pearl	19.90
15778X17_NAK_UPS18-10	Naked Neck	Pearl	21.03
15778X18_TUT_UPS18-13	Turkish Tumbler	Pearl	16.93
15778X19_AUT_UPS17-05	Australian Tumbler	Pearl	19.01
16238X3_VMT_UPS18-15	Vienna Medium Faced Tumbler	Pearl	20.61
16238X4_BLT_UPS12-98	Berlin Long Faced Tumbler	Pearl	19.31
10385X26_MOO_207_B	Mookee	Bull	2.06
10385X27_MOO_316_C	Mookee	Bull	1.44
11573X1_ICE_UPS13-01_barless	Ice Pigeon	Bull	17.05
12097X4_ENT_FB2831_B_F	English Trumpeter	Bull	16.13
12319X4_OGO_UPS15-19_STRWB_M	Old German Owl	Bull	15.93
12319X7_ORF_MDS484_M	Oriental Frill	Bull	15.73
14064X1_COF_UPS16_02_BLU_F	Classic Old Frill	Bull	20.05
14064X2_AFO_UPS16_13_PIE_F	African Owl	Bull	19.89
14064X6_CNT_GN302_M	Chinese Nasal Tuft	Bull	14.57
15321X1_AFO_GN224_WHI_M	African Owl	Bull	24.75
RADDIAAPE_MOO_BGI	Mookee	Bull	20.69
RAHDIAAPE_SCA_BGI	Scandaroon	Bull	18.14
RAUDIAAPE_LAH_BGI	Lahore	Bull	22.69
15778X3_SCM_UPS18-12	Schalkaldener Mohrenkopf	Bull	21.01
15778X4_FAS_UPS14-13	Fairy Swallow	Bull	19.83
15778X14_KOT_MDS511	Komorner Tumbler	Bull	19.06
16238X1_CAN_UPS18-8	Canario Cropper	Bull	21.83
16238X10_SCA_UPS16-12	Scandaroon	Bull	16.13
